# Regulators of H3K4 methylation mutated in neurodevelopmental disorders control axon guidance in *Caenorhabditis elegans*

**DOI:** 10.1242/dev.190637

**Published:** 2020-08-07

**Authors:** Steffen Abay-Nørgaard, Benedetta Attianese, Laura Boreggio, Anna Elisabetta Salcini

**Affiliations:** BRIC, University of Copenhagen, Biotech Research and Innovation Centre, Ole Maaloes vej 5, 2200, Copenhagen, Denmark

**Keywords:** Epigenetics, H3K4 methylation, Neuronal development, Axon guidance, Neurodevelopmental disease, *C*. *elegans*

## Abstract

Post-translational histone modifications regulate chromatin compaction and gene expression to control many aspects of development. Mutations in genes encoding regulators of H3K4 methylation are causally associated with neurodevelopmental disorders characterized by intellectual disability and deficits in motor functions. However, it remains unclear how H3K4 methylation influences nervous system development and contributes to the aetiology of disease. Here, we show that the catalytic activity of *set-2*, the *Caenorhabditis elegans* homologue of the H3K4 methyltransferase KMT2F/G (SETD1A/B) genes, controls embryonic transcription of neuronal genes and is required for establishing proper axon guidance, and for neuronal functions related to locomotion and learning. Moreover, we uncover a striking correlation between components of the H3K4 regulatory machinery mutated in neurodevelopmental disorders and the process of axon guidance in *C. elegans*. Thus, our study supports an epigenetic-based model for the aetiology of neurodevelopmental disorders, based on an aberrant axon guidance process originating from deregulated H3K4 methylation.

## Abstract (precis)

**Summary:** Analysis of mutants lacking many known H3K4 regulators reveals the role of H3K4 methylation in *C. elegans* neuronal functions and suggests that aberrant axon guidance is a shared trait in neurodevelopmental diseases.

## INTRODUCTION

The development of the nervous system requires the coordination of several events, including neuronal progenitor self-renewal, cell migration and differentiation along different lineages, directional neurite outgrowth, and synapse formation. During each step, tight transcriptional control of neurodevelopmental genes is crucial, with chromatin factors playing a major regulatory function by controlling chromatin accessibility (Iwase and Martin, 2018). The contribution of chromatin factors to synaptic plasticity, learning and memory testifies to the broad role of epigenetic mechanisms in the formation and functionality of the nervous system (Ma et al., 2010; Guan et al., 2015; Yao et al., 2016; Kim and Kaang, 2017; Gallegos et al., 2018).

The relevance of chromatin factors in nervous system development is highlighted when considering neurodevelopmental disorders, which are conditions characterized by intellectual disability in which social/motor and learning skills are variably affected (De Rubeis et al., 2014; LaSalle et al., 2013; Pinto et al., 2014; Ronan et al., 2013; Iwase et al., 2017; Gabriele et al., 2018). Advances in next-generation sequencing have allowed a thorough analysis of individuals affected by neurodevelopmental disorders, generating valuable insights for inferring the molecular basis of these diseases. Strikingly, chromatin regulators have emerged as the second most-associated category, outside of genes directly involved in synaptic function (De Rubeis et al., 2014; LaSalle et al., 2013; Pinto et al., 2014; Ronan et al., 2013; Iwase et al., 2017; Gabriele et al., 2018). In particular, regulators of histone 3 lysine 4 (H3K4) methylation are well represented among mutated chromatin factors (Vallianatos and Iwase, 2015; Shen et al., 2014). The levels of H3K4 methylation are dynamically regulated by the action of lysine methyltransferases (KMTs), the majority of which belong to the KMT2 family (KMT2A-D or MLL1-4, and KMT2F/G or SETD1A/B), and lysine demethylases (KDMs) of the KDM1 and KDM5 families (Pedersen and Helin, 2010). KMT2 members are the catalytic subunits of COMPASS-like complexes (complex of proteins associated with Set-1) that include WDR5, RBBP5, ASH2L and DPY30 as core components, and are required for optimal catalytic activity of each complex (Qu et al., 2018; Li et al., 2016).

Mutations in KMT2 members have been identified in cases of Wiedemann–Steine syndrome (Sun et al., 2017; Jones et al., 2012), Kleefstra syndrome (Kleefstra et al., 2012) and Kabuki syndrome (Ng et al., 2010), and are associated with schizophrenia, autism and neurodevelopmental disorders (O'Donnell-Luria et al., 2019; Takata et al., 2016, 2014; Singh et al., 2016). KDM1 and KDM5 members have been found to be mutated in autism spectrum disorders (De Rubeis et al., 2014; Adegbola et al., 2008; Iossifov et al., 2014), X-linked mental retardation (Gonçalves et al., 2014; Abidi et al., 2008; Iwase et al., 2007), non-syndromic intellectual disability (Tunovic et al., 2014; Athanasakis et al., 2014) and Kabuki syndrome (Pilotto et al., 2016; Rauch et al., 2012). Finally, PHF8, a H3K4me3 binder (Tsukada et al., 2010; Kleine-Kohlbrecher et al., 2010; Qi et al., 2010), is altered in cases of X-linked retardation (Redin et al., 2014; Koivisto et al., 2007; Abidi et al., 2007; Laumonnier, 2005; Siderius et al., 1999). Taken together, these results strongly suggest that tight control of H3K4 methylation is crucial for brain development and functionality, and that its deregulation is implicated in the pathogenesis of neurodevelopmental disorders. However, the roles of the H3K4 regulatory machinery in key aspects of neuronal development remain poorly characterized. In particular, how H3K4 methylation impacts axon guidance, a process required to direct the axons to their targets and establish functional neuronal circuits, is unknown. Investigation of this process is limited by the complexity of the mammalian nervous system and by the inadequacy of *in vitro* systems to reproduce physiological conditions. Thus, *in vivo* studies in tractable model organisms could help to dissect the role of histone methylation in this highly conserved biological process (McCammon and Sive, 2015).

*Caenorhabditis elegans*, in which the H3K4 methylation machinery is well conserved, is an amenable model system for studying neurodevelopmental mechanisms. Factors such as a well-defined neuronal connectome and a simple body plan make this organism ideal for unveiling the roles of chromatin factors, and to assess the functional relevance of genetic variations observed in neurodevelopment diseases (Nørgaard et al., 2018; Pedersen et al., 2013; Zallen et al., 1998; Boulin et al., 2006; Adler et al., 2006; Pocock and Hobert, 2008; Johnston and Hobert, 2003). In *C. elegans*, the process of axon guidance can be studied by following the trajectory of PVQ axons (PVQs), which run along the entire animal body in a stereotyped manner. Owing to this invariant pattern of development, the PVQs have been used to identify genes and pathways implicated in axon guidance (Chisholm et al., 2016; Mariani et al., 2016; Riveiro et al., 2017). In this study, we directly tested the role of H3K4 methylation in regulating axon guidance by analysing mutant animals lacking the majority of known H3K4 regulators. The results show that H3K4 methylation regulation is strictly required for the establishment of axon trajectories, and that the deposition of methylation on H3K4 is crucial for neuronal functions related to locomotion and learning.

## RESULTS

### Multiple regulators of H3K4 methylation are required for axon guidance

**Fig. 1.**
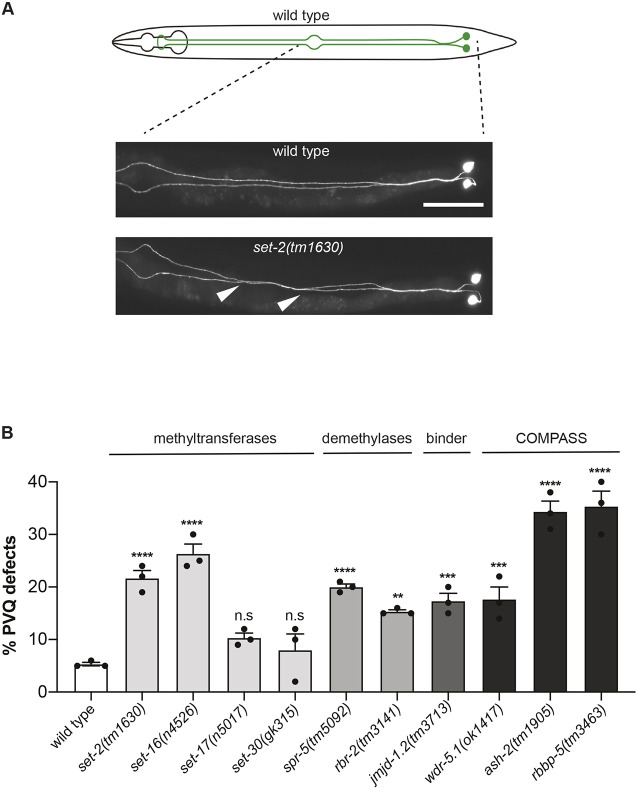
**Loss of H3K4me regulators causes axon guidance defects.** (A) Top: schematic of PVQ neurons in wild type at L4 stage. Bottom: representative image of the posterior section of wild-type and *set-2(tm1630)* L4 worms expressing GFP in PVQ neurons (transgene *oyIs14*). White arrowheads indicate the most common defect observed in mutant animals, in which the left PVQ neuron defasciculates and erroneously migrates to the contralateral side of the ventral nerve cord and back again. (B) Quantification of PVQ defects at L4 stage in wild type and in mutants of genes involved in H3K4me regulation. Mutants for *rbr-2*, a H3K4 demethylase, and *jmjd-1.2*, a H3K4me3 binder, previously reported to exhibit axon guidance defects (Mariani et al., 2016; Riveiro et al., 2017), were used as positive controls. All the alleles used carry large deletions and are most likely null mutants. The *set-16(n4526)* mutant was scored at L1 stage due to larval lethality. *n*>150 for all strains, except for *set-16(n4526)*, *n*=56. Statistical significance testing used one-way ANOVA (Tukey's multiple comparison test). **P<0.005, ***P<0.0005, ****P<0.0001, n.s., not significant compared with wild type. Black dots represent independent scorings. Data are mean±s.e.m. Scale bar: 50 µm.

The PVQs are a pair of interneurons located at the posterior region of the animal, with axons projecting anteriorly during mid-embryogenesis along the ventral nerve cord in two distinct and parallel bundles, which are separated by the ventral midline (Fig. 1A). To test the hypothesis that the regulation of H3K4 methylation is relevant in the establishment of proper axon guidance, transgenic animals expressing a GFP reporter in PVQ neuronal cell bodies and axons were crossed with deletion mutants of components of the H3K4 regulatory machinery. Based on H3K4-related functions (Table 1), we included in our analysis alleles for *set-2*, *set-16*, *set-17* and *set-30*, which were previously reported to act as H3K4 methyltransferases (Fisher et al., 2010; Greer et al., 2014, 2010). We also tested mutant alleles for an H3K4 demethylase, *spr-5* (Nottke et al., 2011), and for genes encoding components of the COMPASS-like complexes (Beurton et al., 2019; Li and Kelly, 2011; Vandamme et al., 2012), such as *wdr-5.1*, *rbbp-5* and *ash-2*. Mutants for the H3K4 demethylase *rbr-2* and for the H3K4 binder *jmjd-1.2* were used as positive controls for phenotypic changes (Mariani et al., 2016; Riveiro et al., 2017). Deletion mutants for *set-2*, *set-16*, *spr-5*, *wdr-5.1*, *ash-2* and *rbbp-5* displayed defects in PVQ axon guidance (Fig. 1B), resulting in aberrant midline crossover of the axons often occurring in the posterior part of the body (Fig. 1A). The axonal defects observed in all mutants were noticeably similar in terms of pattern and penetrance. In contrast, we found that deletions of *set-17* and *set-30* did not compromise the PVQ patterning (Fig. 1B). Interestingly, although human homologues of *set-2* (KMT2F/G), *set-16* (KMT2A-D), *spr-5* (KDM1A), *rbr-2* (KDM5A-D), *jmjd-1.2* (PHF8), *ash-2* (ASH2L), *wdr-5.1* (WDR5) and *rbbp-5* (RBBP5) are mutated in neurodevelopmental diseases (Table 1), no alterations have been reported for the homologues of *set-17* and *set-30* (corresponding to PRDM7/9 and KMT3C, respectively), which were previously reported to methylate H3K4 and H3K36 (Hayashi et al., 2005; Eram et al., 2014; Blazer et al., 2016; Brown et al., 2006; Abu-Farha et al., 2008). Thus, our analysis reveals that the majority of H3K4 methylation regulators in *C. elegans* contribute to the establishment of correct axon guidance, indicating that the regulation of H3K4 methylation is crucial in this process. More importantly, these results highlight a striking and previously unknown correlation between genes regulating H3K4 methylation mutated in neurodevelopmental diseases and genes involved in axon guidance in *C. elegans*.

**Table 1.**
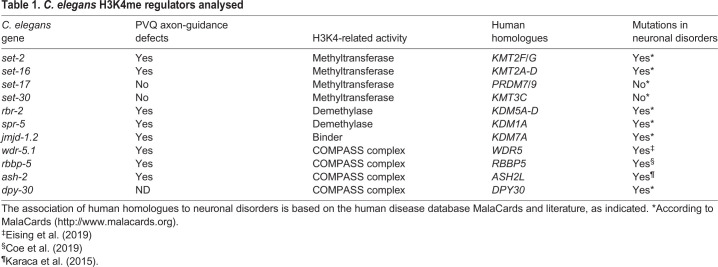
***C. elegans* H3K4me regulators analysed**

### SET-2 controls axon guidance of a subset of neurons

**Fig. 2.**
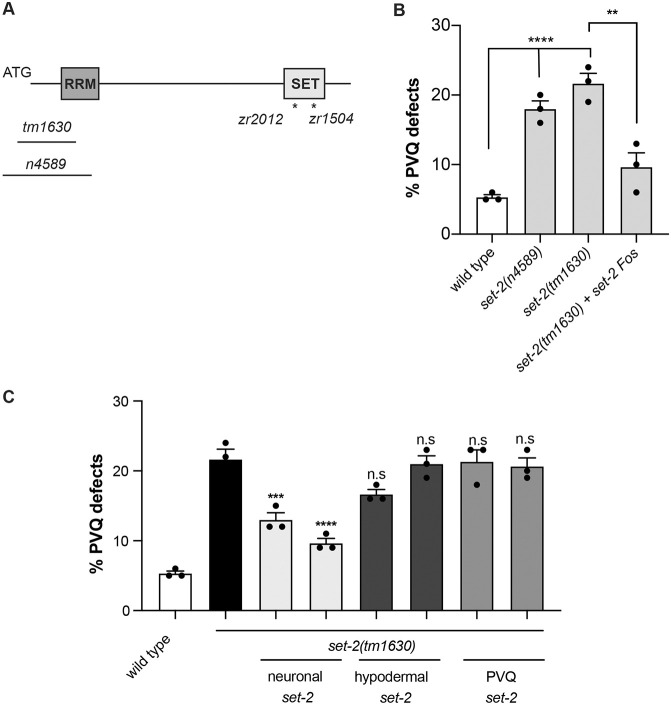
**SET-2 is required in the nervous system to ensure proper PVQ axon guidance.** (A) Schematic of the *set-2* gene. RRM, RNA recognition motif; SET, methyltransferase domain. Deletions and point mutations (indicated by an asterisk) used in this study are shown. (B) Quantification of PVQ defects at L4 stage in wild type and *set-2* deletion alleles, and in *set-2(tm1630)* animals ectopically expressing a genomic region that includes the *set-2* gene (fosmid WRM0638aG05). (C) Quantification of PVQ defects at L4 stage in wild type and *set-2(tm1630)* mutants expressing *set-2* cDNA in different tissues. Promoters used to drive *set-2* expression were as follows: neuronal, *rgef-1*; hypodermal, *dpy-7*; and PVQ, *sra-6*. *n*>150. Statistical significance testing used one-way ANOVA (Tukey's multiple comparison test). *P<0.05; **P<0.005; ***P<0.0005; ****P<0.0001; n.s., not significant. In C, comparisons are with *set-2*. Black dots represent independent scorings. Data are mean±s.e.m.

To gain insight into the molecular mechanisms underlying the axonal defects observed in H3K4 regulator mutants, we characterized the role of *set-2* in detail. *set-2* is homologous to KMT2F/G (also called SETD1a/b), which has essential roles during early mouse embryogenesis (Bledau et al., 2014). In humans, a role for KMT2F/G mutations in neurodevelopmental disorders has been recently suggested by the identification of variants in KMT2F and KMT2G in individuals with intellectual disability, autism, epilepsy and schizophrenia (O'Donnell-Luria et al., 2019; Singh et al., 2016; Hiraide et al., 2018). SET-2 is considered the major methyltransferase for H3K4 in *C. elegans* (Xiao et al., 2011), but its role in neuronal development has not been investigated. The *set-2(tm1630)* and *set-2(n4589)* alleles carry large deletions at the 5′ end of the gene, including the start codon (Fig. 2A), and show similar defective axon guidance phenotypes (Fig. 2B). Furthermore, transgenic expression of a fosmid containing the *set-2* gene in the *set-2(tm1630)* allele rescued the axon guidance phenotype (Fig. 2B). These results strongly suggest that the axonal defect observed is linked to aberrations of *set-2*.

We investigated the focus of action of *set-2* by testing the ability of *set-2* expression in different tissues to rescue the defects observed in *set-2(tm1630)* mutants. Our results showed that SET-2 acts specifically in the nervous system to control PVQ development (Fig. 2C, Fig. S1). However, re-expression of *set-2* in PVQ neurons was not sufficient to rescue the phenotype (Fig. 2B). This result is consistent with a non-cell-autonomous function of *set-2*; however, it should be noted that several technical issues (inappropriate time and/or level of expression) might also account for this negative outcome. To determine whether SET-2 is required in embryos to establish correct axon guidance, or during larval development to maintain PVQ axonal position, we analysed the defect of PVQ axons in freshly hatched larvae. The percentage of axon defects identified in L1 was similar to the one observed in mutant adult animals (16%±2) (Fig. S2), suggesting that SET-2 is required during embryogenesis to ensure proper PVQ axon guidance. In agreement with this, transgenic animals carrying an mCherry-tagged transcriptional reporter showed *set-2* expression in the early embryo (Fig. S3). Loss of *set-2* also impacted the projection of HSN neurons, which extend during larval development, and the axon trajectory of VD and DD neurons in the dorsal nerve cord (Table 2). In contrast, other neurons, such as the mechanosensory neurons (AVM, ALM, PVM and PLM) and the AVK interneuron, displayed normal axon guidance pattern in *set-2* mutant animals (Table 2). Notably, abrogation of *set-2* did not impact the migration of AVM, PVM and HSN neurons (Table 2). These results indicate that SET-2 regulates the projection of several neurons but is not required to organize the overall architecture of the *C. elegans* nervous system.

**Table 2.**
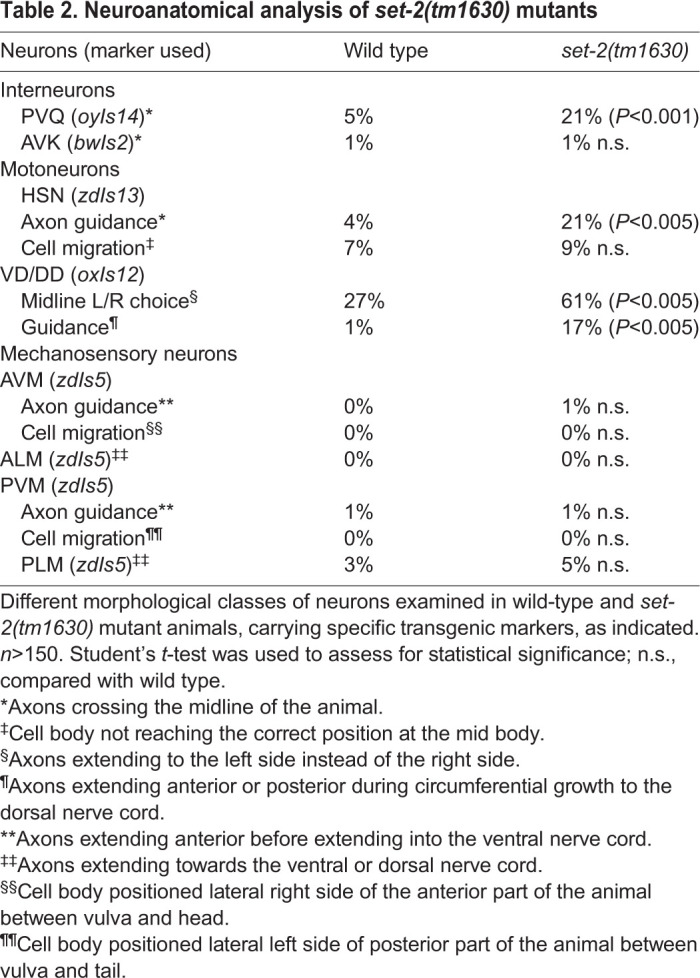
**Neuroanatomical analysis of *set-2(tm1630)* mutants**

### The catalytic activity of SET-2 is required to control axon guidance

**Fig. 3.**
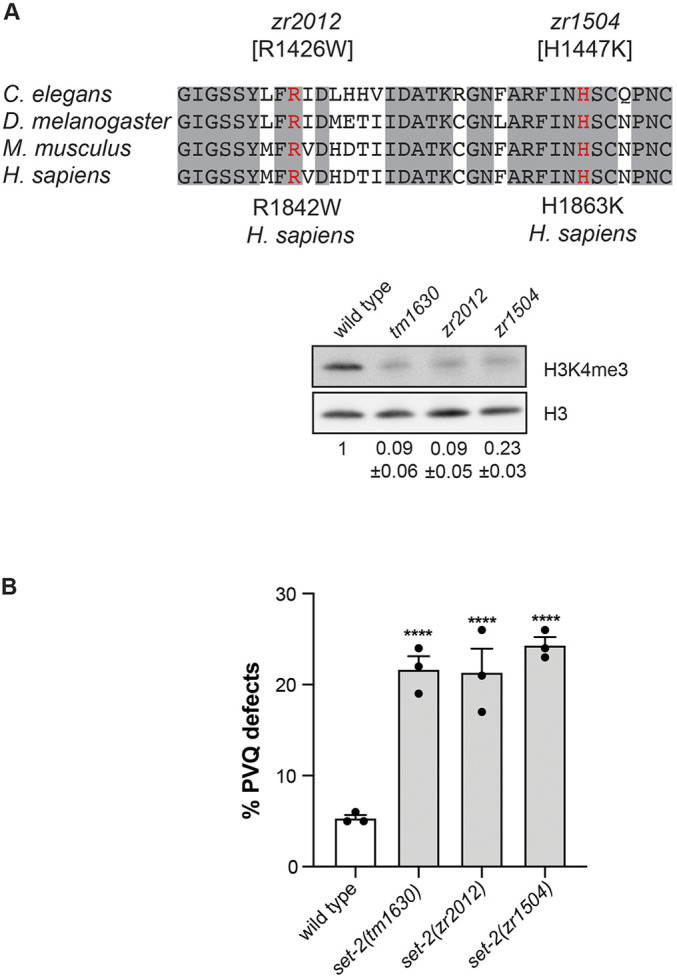
**The catalytic activity of SET-2 is paramount for correct axon guidance.** (A) Top: alignment of a region of the SET domain of SET-2 with homologues in different species. The grey shading denotes conserved amino acids. The red residues denote the conserved amino acids substituted in *set-2* alleles *zr2012* and *zr1504*. Corresponding amino acid substitutions in human are shown. In the *zr2012* allele, Arg1426 is changed to Trp, thus reproducing a disease-associated mutation (R1842W). Bottom: representative western blot showing embryonic H3K4me3 levels in the indicated strains. H3 was used as loading control. The numbers indicate the average of H3K4me3 relative to wild type, from three independent experiments ±s.e.m. (B) Quantification of PVQ defects at L4 stage in wild type and indicated *set-2* mutant alleles. Statistical significance testing used one-way ANOVA (Tukey's multiple comparison test). *n*>150, ****P<0.0001 compared with wild type. Black dots represent independent scorings. Data are mean±s.e.m.

SET-2 mainly catalyzes the tri-methylation of H3K4 (Li and Kelly, 2011; Xiao et al., 2011). In agreement with this, we observed strongly reduced levels of H3K4me3 in the *set-2(tm1630)* mutant embryos, indicating that SET-2 is the main enzyme catalyzing H3K4me3 in embryos (Fig. 3A, Fig. S4). Several point mutations in the SET domain have been shown to perturb the activity of the protein without compromising its stability (Rickels et al., 2017; Dorighi et al., 2017). To directly assess the relevance of the enzymatic activity of SET-2, and therefore of H3K4me3, in the context of axonal guidance, we introduced mutations in the *set-2* gene giving rise to two mutated alleles *set-2(zr1504)* and *set-2(zr2012)*, in which conserved amino acids located in the SET domain were mutated (H1447K and R1426W, respectively, Fig. 3A). In the *set-2(zr2012)* allele, we introduced a mutation leading to the same amino acid substitution found in SETD1B/KMT2G in a case of intellectual disability linked to epilepsy and autism (Hiraide et al., 2018). Therefore, the *set-2(zr2012)* allele provided a simple model with which to test the effects of a disease-associated mutation of *set-2* in an *in vivo* context. In both mutant animals, we observed a strong reduction of H3K4me3 levels, similar to the one detected in the *set-2(tm1630)* deletion allele, both by western blot and immunofluorescence in embryos (Fig. 3A, Fig. S4). Importantly, these mutant alleles showed axonal defects with similar penetrance to the *set-2(tm1630)* deletion mutant (Fig. 3B). Therefore, our result, together with the evidence (Fig. S5) that no axon abnormalities are observed in the *set-2(ok952)* allele, an in-frame deletion in which the levels of H3K4me3 in embryos were not affected (Xiao et al., 2011), suggests the catalytic activity of SET-2 is crucial for proper axon guidance.

### SET-2 genetic interactions with pathways regulating axon guidance

Multiple conserved redundant pathways control axon guidance. The role of several signalling pathways like Netrin, Slit, Ephrins and Semaphorin in this context is well characterised in *C. elegans*. Similarly, the relevance of transmembrane proteins such as Syndecan and other proteoglycans is well established (Chisholm et al., 2016). Genetic interaction assays have been used to determine the components of these pathways and to establish functional relationships among the genes involved (Zallen et al., 1998; Bülow et al., 2008). In order to assess whether *set-2* acts within known pathways, we generated animals carrying the *set-2(tm1630)* allele together with mutations of genes belonging to the major axon guidance pathways, and analysed the trajectories of the PVQ neurons. Concomitant abrogation of *set-2* and components of the Ephrin (*vab-1*) or Semaphorin (*plx-2*) pathways resulted in a phenotype that had a penetrance similar to the one observed in the single mutants (Table 3). Analogous results were obtained in *sdn-1;set-2*. On the contrary, when the Netrin (*unc-5*) and *sax-3*/ROBO pathways (*sax-3*) were ablated in the *set-2* genetic background, we observed an exacerbation of the phenotype (Table 3). Therefore, *set-2* appears to act in parallel with the Netrin and SAX-3/ROBO pathways, and in concert with Ephrins and Semaphorin, the main antero-posterior signalling pathways involved in axon guidance.

**Table 3.**
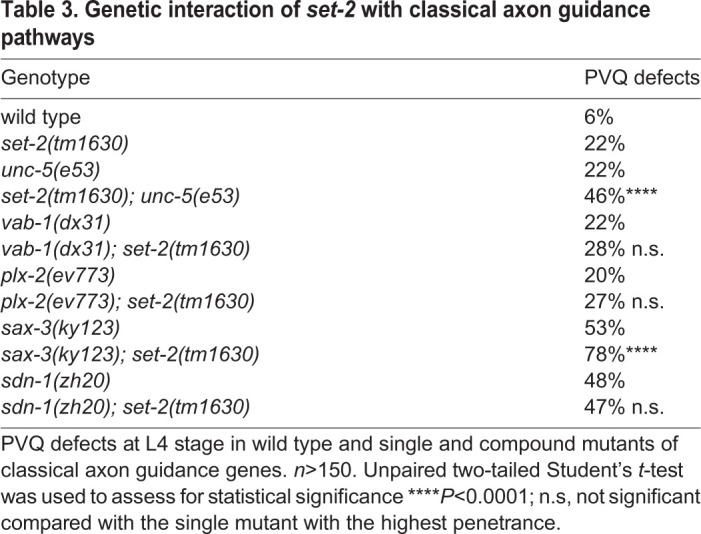
**Genetic interaction of *set-2* with classical axon guidance pathways**

**Fig. 4.**
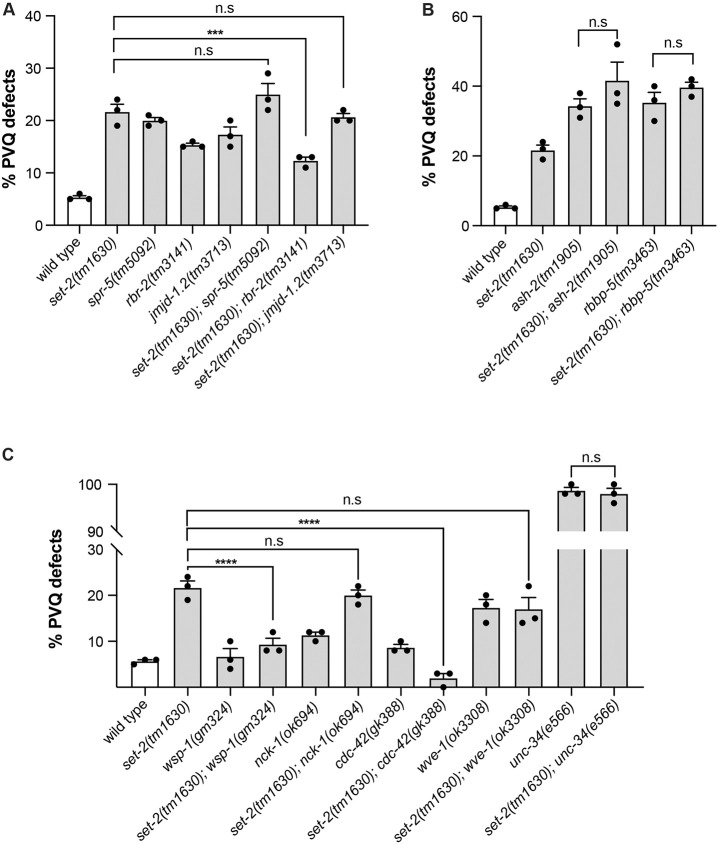
***set-2* genetic interactions with other H3K4me regulators and actin modulators.** (A) Quantification of PVQ defects at L4 stage in wild type, and in single and compound mutants of H3K4 regulators. (B) Quantification of PVQ defects at L4 stage in wild type, and in single and compound mutants of COMPASS complex members. (C) Quantification of PVQ defects at L4 stage in wild type and single and compound mutants of actin modulators. *n*>150 for all strains [except for *cdc-42(gk388); set-2(tm1630)*, *n*=63]. Statistical significance testing used one-way ANOVA (Tukey's multiple comparison test). ***P<0.0005, ****P<0.0001 n.s., not significant compared with single mutants with the highest penetrance. Black dots represent independent scorings. Data are mean±s.e.m.

A similar experimental approach was performed to analyse the crosstalk among the H3K4 methylation regulators we found involved in axon guidance. To investigate the relationship of *set-2* with *rbr-2*, *spr-5* and *jmjd-1.2*, we analysed the PVQ defects of animals lacking a combination of these genes. None of the double mutants showed an additive effect (Fig. 4A), suggesting that the regulators act jointly to ensure the correct levels of H3K4 methylation and normal axon guidance. However, the abrogation of *rbr-2* in the *set-2(tm1630)* background led to an amelioration of the axon phenotype, suggesting that *rbr-2*, likely through its H3K4 demethylase activity, can counteract the effect of *set-2* in axon guidance. A similar neutralizing effect of *rbr-2* mutations has been observed previously for the lifespan phenotype of *set-2* (Greer et al., 2010). Finally, we analysed the penetrance of the defects in compound mutants of *set-2* with *rbbp-5* or *ash-2*, components of the COMPASS complexes. Double mutants showed levels of defects similar to those observed in single mutants (Fig. 4B), suggesting that *set-2* controls the axon guidance process in the context of the COMPASS complex.

### *set-2* controls axon guidance by regulating actin remodelling through *wsp-1*

Actin remodelling at the growth cone is ultimately the key process directing axon guidance. We therefore tested whether the defect observed in *set-2* mutant animals could be related to aberrant regulation of actin dynamics. We generated double mutants eliminating *set-2* in concomitance with *wsp-1/WASP*, *wve-1/WAVE* and *unc-34/Ena/VASP*, which are known actin-regulator genes (Higgs and Pollard, 2001). Although we observed no effect with *unc-34* or *wve-1*, ablation of *wsp-1* fully rescued the *set-2* axon guidance phenotype (Fig. 4C). This result suggests that *set-2* controls axon guidance by regulating actin remodelling specifically through *wsp-1*. We also tested the effect of *cdc-42* and *nck-1* ablation, the mammalian homologues of which are known activators of N-WASP (Alekhina et al., 2017). As only loss of *cdc-42* rescued the *set-2* phenotype (Fig. 4C), we conclude that an aberrant CDC-42-dependent activation of WSP-1 is likely fundamental to the axonal defect observed in *set-2* mutant animals.

### Transcriptional regulation mediated by SET-2

**Fig. 5.**
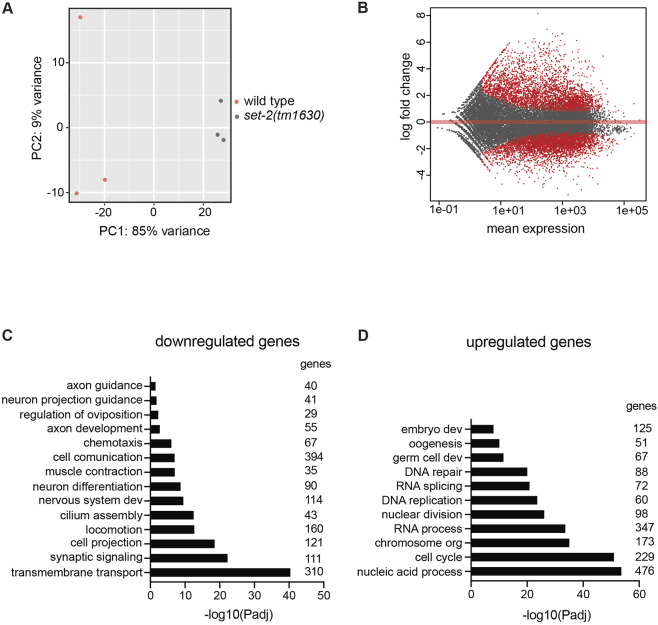
**Transcriptional regulation mediated by SET-2.** (A) PCA plot of wild-type and *set-2(tm1630)* mid-embryos. Each dot represents one sample and each colour a genotype. (B) MA plot showing gene expression changes in *set-2(tm1630)*. The *x*-axis represents the mean of counts, the *y*-axis represents log2 fold change. DE genes with FDR<0.05 are shown in red. (C,D) GO analysis of the biological processes of downregulated (C) and upregulated (D) genes in the *set-2(tm1630)* allele using g:Profiler and adjusted *P*-values (Bonferroni correction). Selected top scoring categories are presented together with the number of genes identified in each category.

H3K4me3 is a post-translational modification identified at promoter regions of transcriptionally active genes and, in agreement with this, loss of *set-2* has been reported to deregulate transcriptional activity (Beurton et al., 2019; Robert et al., 2014). To gain insight into the mechanisms of action of SET-2 in axon guidance, we analysed the transcriptome of *set-2(tm1630)* mutants at the mid-embryonic stage in which PVQ axon development occurs. Principal component analysis (PCA) of RNA-sequencing datasets from wild-type and *set-2(tm1630)* animals indicated that the gene expression patterns in *set-2* mutant embryos were significantly different from wild-type embryos (Fig. 5A), with 6444 genes (FDR<0.05) differentially expressed (DE) (Fig. 5B, Table S1). A similar number of genes were downregulated and upregulated in comparison with wild-type animals (Table S1). The median log2 fold changes of gene expression were 2.27±0.018 (mean±s.e.m.) for upregulated genes and 1.78±0.01 (mean±s.e.m.) for downregulated genes. Strikingly, among the downregulated genes, gene ontology (GO) analysis identified genes associated with neuronal function categories, including neuronal development, locomotion, chemotaxis, neuronal cell projection, axon guidance and synaptic transmission (Fig. 5C,Table S1). And, with the exception of *sax-3*, all the other genes involved in axon guidance pathways tested for genetic interactions are listed as downregulated genes in the RNA-sequencing dataset. Among the upregulated genes, categories related to germ cell biology and DNA replication/repair were significantly enriched (Fig. 5D), corroborating previous studies that implicated *set-2* in fertility and genome stability (Xiao et al., 2011; Herbette et al., 2017). We also analysed the transcriptome of the *set-2(zr2012)* allele that expresses a mutant SET-2 protein with an amino acid substitution found in SETD1B in a case of intellectual disability (Hiraide et al., 2018). Despite a smaller number of DE genes identified in animals carrying this allele (3053 DE genes, FDR<0.05) (Table S1, Fig. S6), the intersection of DE genes and consistently downregulated genes in the two *set-2* alleles was significant (*P*<0.001 and *P*<0.0001, respectively). Importantly, GO analysis of downregulated genes in *set-2(zr2012)* and consistently downregulated genes in *set-2(tm1630)* and *set-2(zr2012)* identified neuronal categories as enriched, confirming the relevance of *set-2* in positively regulating the transcription of neuronal genes (Fig. S6, Table S1). These results suggest that, in agreement with its catalytic activity, SET-2 contributes substantially to the regulation of gene expression in embryos. Furthermore, the identification of several downregulated genes belonging to the cell projection/axon guidance class corroborates our finding that the regulation of H3K4 methylation is required for the establishment of proper axon trajectory.

### Loss of *set-2* impairs nervous system functionality

**Fig. 6.**
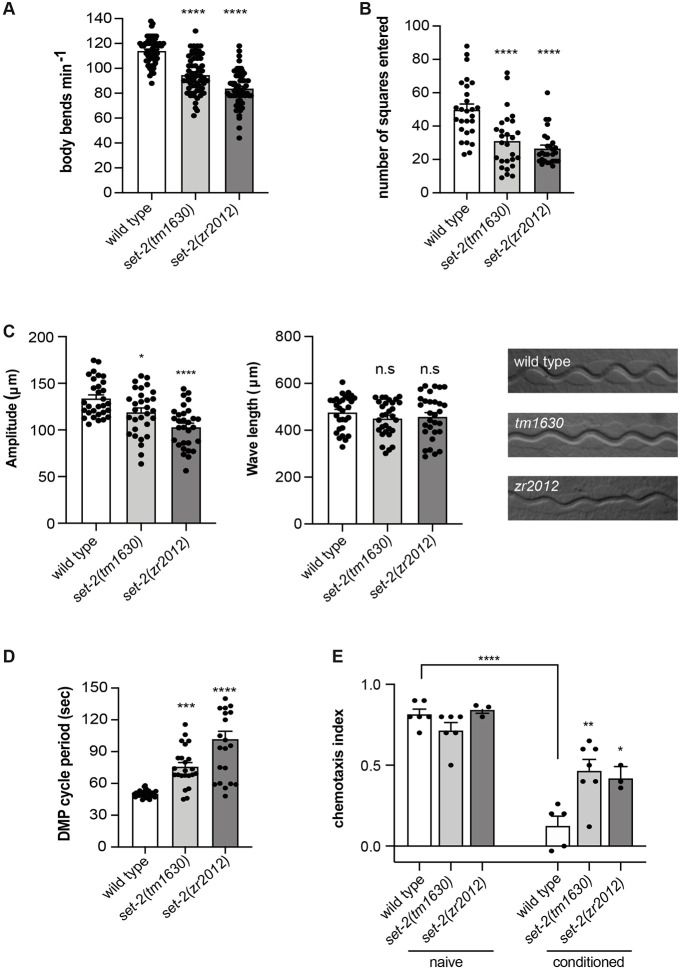
**Loss of SET-2 impairs** **nervous system functionality.** (A) Quantification of body bends in liquid in wild type, *set-2(tm1630)* and *set-2(zr2012)*. *n*>60, Student's *t*-test. (B) Quantification of exploratory behaviour in wild type, *set-2(tm1630)* and *set-2(zr2012)*. *n*>30, Student's *t*-test. (C) Left and middle: quantification of wavelength and amplitude of tracks left on a bacterial lawn by wild type, *set-2(tm1630)* and *set-2(zr2012)*. *n*>30, Student's *t*-test. Right: representative images of crawling tracks in wild-type, *set-2(tm1630)* and *set-2(zr2012)* mutant animals. (D) Quantification of DMP length in wild type, *set-2(tm1630)* and *set-2(zr2012)*. *n*>30, Student's *t*-test. (E) Quantification of the chemotactic response to NaCl and plasticity in *set-2(tm1630)* and *set-2(zr2012)*. Worms were washed and placed directly onto assay plates (naive) or onto conditioning plates for 1 h before being tested (conditioned). Chemotactic indices of mutant strains in the conditioned state were compared with wild type in the same conditions. The comparison of chemotactic indices of wild type in naive and conditioned states is marked with a line. *n*>200, Statistical significance testing used one-way ANOVA (Tukey's multiple comparison test). ****P<0.0001, ***P<0.001, **P<0.01, *P<0.05, n.s., not significant compared with wild type. In A-D, black dots represent single animals. In E, black dots represent independent replicates. Error bars indicate s.e.m.

Besides the genes required for the establishment of proper axon guidance, the transcriptome analysis of *set-2* mutant alleles revealed that numerous neuronal genes were differentially expressed, suggesting a broad role for *set-2* in the nervous system. Therefore, we tested whether the loss of *set-2* would result in abnormal neuronal functionality. Despite *set-2(tm1630)* and *set-2(zr2012)* mutant animals appearing superficially wild type, we detected differences when compared with control animals in specific functional tests. Locomotion in *C. elegans* is controlled by excitatory cholinergic and inhibitory GABAergic motor neurons, the functionality of which can be determined by observing animals swimming in liquid. *set-2(tm1630)* and *set-2(zr2012)* mutant animals displayed a reduced rate of body bends in liquid compared with wild-type animals (Fig. 6A). Furthermore, when left moving on a plate for 1 h at 20°C, *set-2(tm1630)* and *set-2(zr2012)* mutants appeared to explore fewer regions of the plate (Fig. 6B) and to move in a tighter circular pattern compared with control animals (Fig. S7). A closer analysis of the crawling tracks revealed differences in wave amplitude between control animals and mutants, but no significant differences were observed when comparing wave lengths (Fig. 6C). Overall, these results suggest abnormal locomotion behaviour in *set-2(tm1630)* and *set-2(zr2012)* mutant animals. Defecation in *C. elegans* is the result of a stereotyped and tightly regulated motor programme involving the subsequent contraction of three distinct sets of enteric muscles (Schuske et al., 2004). Monitoring this relatively simple process is a powerful method for determining neuronal system functionality and synaptic transmission. We found that the rate of defecation in both *set-2* mutants was significantly reduced compared with wild-type animals (Fig. 6D). Last, we assessed chemotaxis responses towards attractive stimuli (Mori, 1999) by testing the response of *set-2* mutant animals to sodium chloride. No differences were observed in the *set-2(tm1630)* and *set-2(zr2012)* mutant animals compared with control animals, suggesting that *set-2* mutants have an intact chemotactic response (Fig. 6E). In *C. elegans*, the chemotactic response changes according to previous experiences (Tomioka et al., 2006; Saeki et al., 2001). When animals are grown in the presence of food and sodium chloride, they are attracted to the salt. In contrast, when worms are starved in the presence of salt, they learn to avoid it as they associate the salt with an unfavourable condition. We tested whether this associative learning process was affected in *set-2(tm1630)* and *set-2(zr2012)* mutant animals by conditioning animals in unseeded plates containing sodium chloride and subsequently testing their reaction to the salt. In contrast to wild-type animals, conditioned *set-2* mutants were still attracted to sodium chloride, suggesting a defect in the associative learning process (Fig. 6E). Overall, these results indicate that mutations in the *set-2* gene, in correlation with aberrant expression of neuronal genes, result in compromised neuronal functions.

## DISCUSSION

Despite the recognition of H3K4 methylation as a crucial epigenetic modification in neuronal development, its biological role in this tissue is only marginally understood (Mariani et al., 2016; Riveiro et al., 2017). In this study, we specifically addressed the role of the main H3K4 methyltransferase SET-2 in neuronal development using *C. elegans* as a model system. Our results demonstrate the requirement of the catalytic activity of SET-2, and therefore for H3K4 methylation, in the process of axon guidance. Moreover, we showed that several proteins involved in the regulation of H3K4 methylation are also required for the establishment of axon trajectories, including *set-16*, another H3K4 methyltrasferase. We do not know whether *set-2* and *set-16* have a redundant role in axon guidance and share common targets. Nevertheless, the requirement of multiple H3K4 regulators likely reflects the notion that axon guidance is a complex process regulated by a multitude of extracellular cues and signalling pathways that need to be integrated, and temporally and spatially coordinated. We propose that such orchestration is, at least in part, epigenetically controlled and occurs by fine-tuning the transcription of the implicated genes through H3K4 methylation regulation.

**Fig. 7.**
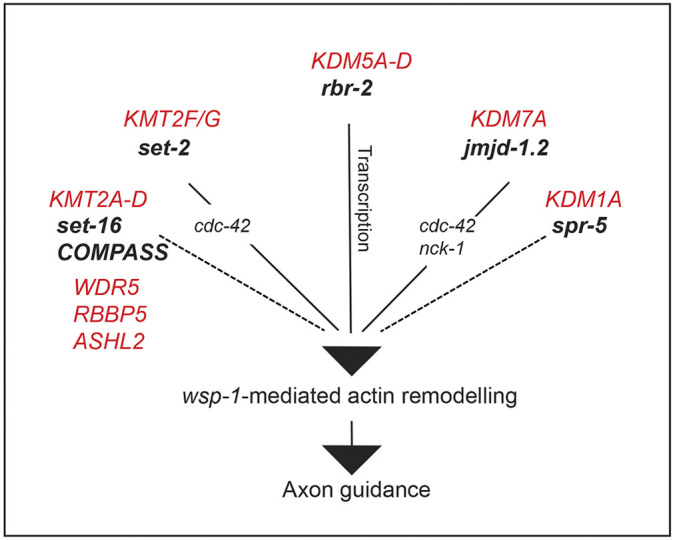
***C. elegans* homologues of H3K4 regulators mutated in disease all contribute to axon guidance.** Schematic model depicting the role of *rbr-2*, *set-2* and *jmjd-1.2* in regulating *wsp-1*-mediated actin remodelling and axon guidance. *rbr-2* directly influences *wsp-1* transcription (Mariani et al., 2016), whereas *set-2* and *jmjd-1.2* control *wsp-1* activity through *cdc-42* and *nck-1* (this study and Riveiro et al., 2017). The action of *set-16*, COMPASS complex components and *spr-5* on *wsp-1* remain to be elucidated. Mammal homologues found mutated in neurodevelopmental diseases are shown in red.

Our analysis showed that the catalytic activity of SET-2 is required for the correct axon guidance through a mechanism that involves the regulation of cytoskeleton dynamics. Remarkably, the axon guidance defects observed in the H3K4 methylation regulators investigated in detail so far (*rbr-2*, *jmjd-1.2* and *set-2*) are all suppressed by *wsp-1* ablation (this study and Mariani et al., 2016; Riveiro et al., 2017), suggesting that, in line with the genetic interaction observed among *set-2*, *rbr-2* and *jmjd-1.2*, *wsp-1* is a shared target (Fig. 7). Our results, showing that only *wsp-1*, and not other known actin regulators like *unc-34* or *wve-1*, suppresses the axonal defects, suggests that the H3K4 regulatory machinery controls a specific branch of actin remodelling. It should also be noted that the modalities by which H3K4 regulators control WSP-1 functionality are likely different, as *rbr-2* controls *wsp-1* expression (Mariani et al., 2016), whereas *jmjd-1.2* appears to regulate its activation through *nck-1* and *cdc-42* (Riveiro et al., 2017). The effect of *set-2* on *wsp-1* activation seems to strictly depend on *cdc-42*, as ablation of *cdc-42*, but not *nck-1*, ameliorates the axonal phenotype. Puzzlingly, in *set-2(tm1630)*, the levels of *wsp-1* expression appeared reduced (Table S1). As loss of *wsp-1* did not result in axon defects, the *wsp-1* downregulation observed is likely due to a compensatory mechanism that reduces the effects of its aberrant activation. The identification of direct targets of SET-2 will help to clarify the specific mechanism underlying the genetic interactions observed.

It is intriguing that only a subset of neurons is invariantly disturbed by the loss of H3K4 regulator genes (this study and Mariani et al., 2016; Riveiro et al., 2017), suggesting that in these neurons the process of axon guidance is particularly vulnerable and under epigenetic control. This possibility is also suggested by a study showing that defects in axon guidance of the same subset of neurons are observed in animals experiencing oxygen deprivation during embryonic development (Pocock and Hobert, 2008). Further analyses testing other adverse environmental conditions, and cell-specific studies related to expression patterns and lineage, will help us to understand the origin of this susceptibility.

*set-2* mutant alleles show phenotypes related to locomotion and learning. It is likely that these phenotypes might only partially depend on axon-guidance defects. Indeed, transcriptional profiles and GO analyses of *set-2* alleles identified several neuronal categories among DE genes, which suggest a novel and broad role for *set-2* in the *C. elegans* nervous system. The role of *set-2* mammalian homologues in neurons has not been investigated owing to the essential nature of these genes during mouse embryogenesis. However, considering our results and the frequency of mutations in these genes in cases of intellectual disability, autism, epilepsy and schizophrenia (O'Donnell-Luria et al., 2019; Singh et al., 2016; Hiraide et al., 2018), it is tempting to hypothesise an evolutionarily conserved role for KMT2F and KMT2G in nervous system development, which deserves further investigation.

In the context of disease, the recent identification by genome-wide association studies of a H3K4 regulation domain affected in neuronal disorders has emphasised the potential impact of epigenetic regulation in the developing nervous system and in illness. By demonstrating that *C. elegans* homologues of H3K4 regulators mutated in disease are all required for proper axon guidance (Fig. 7), and that a disease-associated mutation of SETD1B reproduces the axonal phenotype in the nematode, our studies provide evidence for a common denominator among these genes. Altogether, our results suggest that aberrant axon guidance is a shared trait in neurodevelopmental diseases and offer experimental support to a recently suggested hypothesis proposing that dysregulated axonal guidance underlines neurological disorders (Van Battum et al., 2015; Niftullayev and Lamarche-Vane, 2019; McFadden and Minshew, 2013).

## MATERIALS AND METHODS

### Genetics and strains

*C. elegans* strains were cultured using standard growth conditions at 20°C with *Escherichia coli* OP50 (Brenner, 1974). Double mutant animals were generated by using a standard crossing procedure. *set-2(tm1630)* was backcrossed six times and *set-2(zr2012)* was backcrossed two times. Neuronal marker strains were backcrossed three times. The strains used are listed in Table S2. *set-16(n4526*) is a balanced strain. Heterozygotes segregate Dpy sterile animals (+/+), larval lethal animals (*−/−*) and wild-type animals (+/−).

### Generation of constructs

The *set-2* transcriptional reporter includes a ∼400 bp region located at the 5′ end of the *set-2* gene amplified using the following primers: 5′-ccgatgcacagtagaaatctg-3′ and 5′-gcaaacttcatatccagaccata-3′. The PCR product was cloned into pD95.75mCherry. Tissue-specific rescue constructs were made using a MultiSite Gateway Three-Fragment Vector Construction Kit (Life Technologies) as described previously (Mariani et al., 2016). The *set-2* cDNA was a kind gift from Francesca Palladino (École normale supérieure de Lyon, France).

### Generation of transgenic lines

Transgenic lines were created by microinjection (Mello et al., 1991). The *set-2* transcriptional reporter line was obtained by injecting 50 ng/µl of reporter construct into the N2 strain. Tissue-specific rescue constructs (10 ng/µl) were injected along with *myo-2*::mCherry (5 ng/µl) as a co-injection marker into *set-2(tm1630)*. The fosmid (5 ng/µl) was injected along with co-injection marker *myo-2*::mCherry (5 ng/µl). The fosmid (WRM0638aG05) was a kind gift from Roger Pocock (Monash University, Melbourne, Australia). The transgenic lines used in this study are listed in Table S2.

### CRISPR lines

CRISPR lines were created by injecting ssDNA repair templates for *set-2* with desired substitutions cloned into pJJR50 (*zr1504* sgRNA sequence CCTTCGCGTAGCAATTAGGT and *zr2012* sgRNA sequence TCACATGATGCAGATCAATT). The mix contained a *pha-1* repair template and pJW1285 (driving the expression of Cas9) was injected into *pha-1(e2123)* mutants. All constructs were injected at a concentration of 50 ng/µl. Selection for *pha-1* wild-type clones was performed at 25°C. Mutations were confirmed by sequencing. The mutation in the *zr1504* allele was selected based on the following criteria: (1) conserved from yeast to human; (2) outside of the interaction surface with other components of the mixed lineage leukaemia-complex based on Shinsky et al. (2014); and (3) a conservative substitution (H to K). The mutation in the *zr2012* allele reproduces an alteration identified in SETD1B/KMT2G in a case of intellectual disability linked to epilepsy and autism (Hiraide et al., 2018).

### Western blot and immunostaining

Protein extracts were prepared from embryos obtained after hypochlorite treatment of animals grown at 20°C. Samples were boiled in SDS-PAGE buffer for 5 min and sonicated for 10 min using a Diagenode Bioruptor (UCD-300). The following antibodies were used: anti-H3K4me3 (Cell Signaling Technology, C42D8; 1:750); polyclonal anti-H3 (Abcam, ab1791; 1:10.000); and peroxidase-labelled anti-rabbit secondary antibody (Vector Laboratories; 1:10.000). Western blots were quantified using ImageJ [National Institutes of Health (NIH)].

Embryo staining was performed as described by Chin-Sang et al. (1999). The primary antibody for H3K4me3 (Cell Signaling Technology, C42D8; 1:500) was incubated overnight at 4°C and the secondary antibody [goat anti-rabbit IgG (Alexa Fluor 488, Invitrogen, A11008; 1:500)] was incubated for 2 h at room temperature. Embryos were stained with DAPI and slides were mounted using mounting media.

### Axon guidance analyses

The axon guidance phenotype was scored at 20°C at the L4 stage, unless otherwise stated. Worms were immobilized in NaN_3_ and placed on microscope slides with a 5% agarose pad. Results from at least three biological independent experiments were used for statistical analyses. Images were obtained using a Zeiss AXIO Imager M2 fluorescence microscope. Owing to the larval lethality of the strain, the scoring of the *set-16* mutant was conducted at the early larval stage. Arrested larvae were considered *set-16* null. Investigators were not blinded during the experiments.

### Statistical analyses

Statistical analysis for all neuronal scoring was performed using GraphPad Prism 8 using Student's *t*-test or one-way ANOVA (Tukey's multiple comparison). All values are presented as mean percentages.

### RNA-sequencing

RNA was isolated from three independent experiments per genotype. Hermaphrodites were bleached twice to achieve better synchronization. Eggs recovered from the second hypochlorite treatment of highly synchronized young adult animals were kept at 20°C for 4 h in M9 media to reach mid-embryogenesis (the majority of the eggs were at comma stage) and freeze cracked in liquid nitrogen. Wild-type and *set-2* samples were prepared and analysed in parallel, to minimise, as much as possible, synchronisation and batch issues. RNA was extracted using an Arctutus PicoPure RNA Isolation Kit (Thermo Fisher Scientific, KIT0204). Sequencing libraries was constructed using a TruSeq RNA Library Prep Kit v2 (Illumina, RS-122-2001). Libraries were sequenced using a NextSeq 500 system and a NextSeq 500/550 High Output Kit v2 (Illumina, FC-404-2005).

### RNA-sequencing analysis

RNA-sequencing results were analysed using Galaxy (v19.05). Reads were mapped to the C. *elegans* genome (WS220) using a criterion of two mismatches. Number of reads aligned for each replica was between 14.6 to 53.1 million. DESeq2 (v2.1.8.3) was used to determine DE genes and to generate principal component and scatter plots. DE genes with FDR<0.05 were analysed using g:Profiler (biit.cs.ut.ee/gprofiler/gost) with Bonferroni correction. The *P* value for overlapping gene lists was calculated using the statistical significance of the overlap between two groups of genes tool (www.nemates.org/MA/progs/overlap_stats.html).

### Neuronal function analyses

#### Thrashing assay in liquid

A 96-well microtitre plate, with each well containing 400 µl of M9 media, was used. Three young adult-stage worms of the same strain were placed in each well and left for 10 min at 20°C to adapt. Body bends were counted for 30 s. One bend was counted every time the mid-body of the animal returned to the same position. The experiment was carried out using at least 60 worms per strain.

#### Tracking and exploration assay

Nematode Growth Media (NGM) plates (6 cm) were seeded with 600 µl of OP50 grown overnight in lysogeny broth at 37°C and stored at 25°C for one night. One young adult stage worm was placed in the centre of the bacteria lawn and left to crawl for 1 h at 20°C. For the tracking assay, animals were removed after 1 h of crawling and the body length was measured for normalization. The tracks that the animals left on the plates were visualized using a dissecting microscope and a digital camera using the same magnification settings (20×). The amplitude and wavelength of the tracks were measured using ImageJ. For the exploration assay, animals were removed after 1 h of crawling and plates were superimposed on a grid containing 3×3 mm wide squares, and the number of squares entered by the worm were manually counted as described previously (Juozaityte et al., 2017). Both assays were performed using at least 30 worms per strain.

#### Defecation assay

Defecation was assessed as described previously (Mahoney et al., 2008). Each defecation motor program (DMP) cycle was counted as the interval between two posterior body-wall muscle contractions. Five full cycles for each animal were counted. This assay was carried out using at least 30 worms per strain.

#### Chemotaxis and chemotaxis plasticity assay

The chemotactic response to NaCl was conducted as described previously (Tomioka et al., 2006). Briefly, 20 ml of buffered agar was poured into 10 cm diameter Petri dishes. To set up a salt gradient, 10 µl of 2.5 M NaCl solution was applied to the attractant spot, and 10 µl of ddH2O was applied to the control spot 16 h before the assay. Another 10 µl of 2.5 M NaCl solution or water was applied 4 h before the assay onto the same spots. NaN_3_ (1 µl) was applied to both spots 1 min before the assay. Synchronized young adult animals were washed three times with chemotaxis (CTX) solution [5 mM KH_2_PO_4_/K_2_HPO_4_ (pH 6), 1 mM CaCl_2_ and 1 mM MgSO_4_], and 40 to 50 worms were placed in the centre of the assay plate in a minimal volume buffer. Animals were left to crawl for 45 min at 20°C, after which the plates were placed at 4°C overnight and the chemotactic index was calculated. The chemotaxis index was defined as the number of animals in the NaCl area (within 1.5 cm of the solution spot) minus the number of animals in the control area, divided by the total number of animals on the plate. Worms unable to leave the centre of the assay plate were censored. The chemotaxis was assessed with assay plates prepared in the same way as above. Synchronized young adult animals were washed three times with CTX solution, and 40 to 50 worms were placed onto conditioning plates prepared with NGM media (containing NaCl) and without *E*. *coli* OP50. Animals were conditioned for 1 h, washed again once with CTX solution and placed in the centre of the assay plates. Worms were left to crawl for 45 min at 20°C, after which the plates were placed at 4°C overnight. The chemotactic index was calculated as before. Several independent biological replicates were analysed. Investigators were not blinded during the experiments.

## Supplementary Material

Supplementary information

Reviewer comments
