# Supplementary material for: Regulators of H3K4 methylation mutated in neurodevelopmental disorders control axon guidance in Caenorhabditis elegans
Source: Development. 2020 Aug 7;147(15):dev190637. doi: 10.1242/dev.190637 (PMC7420840; doi:10.1242/dev.190637)
Supplement: Supplementary information [file develop-147-190637-s1.pdf]

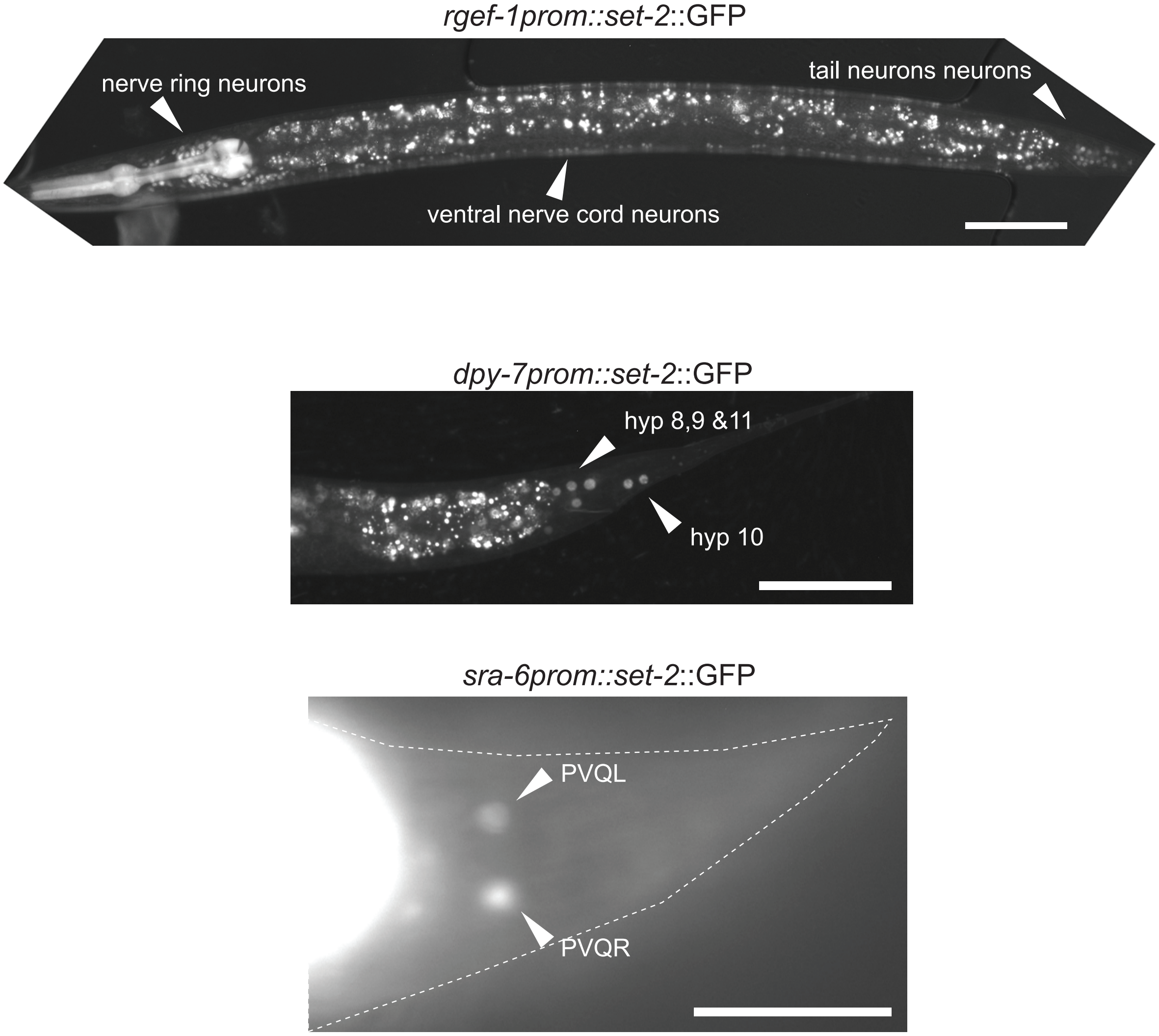

Figure S1. Expression of transgenic SET-2::GFP in specific tissues. Top: Expression pattern of SET-2::GFP under the control of the neuronal specific *rgef-1* promoter. Pharynx is visible as *myo*-2::mCherry was used as co-injection marker. Middle: Expression pattern of SET-2::GFP under the control of the hypodermal specific *dpy-7* promoter. Depicted is the posterior of the animal. Bottom: Expression pattern of SET- 2::GFP under the control of the PVQ promoter *sra-6*. The posterior extremity of the animal is outlined with white-dotted line. Strong fluorescence in the left of the picture is autofluorescence from the gut. Scale bars: 50 μm.

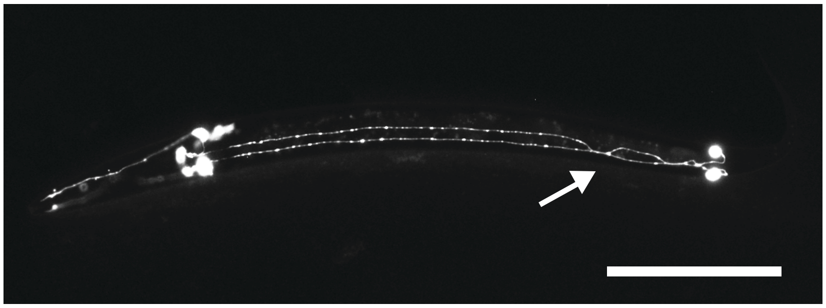

Figure S2: Axon guidance defect in *set-2(tm1630)* at L1 stage. Representative image of a *set-2(tm1630)* L1 carrying the *oyIs14* transgene. Arrow indicates an aberrant pattern of PVQ axon guidance in the posterior part of the animal. Scale bar: 50μm.

Development • Supplementary information

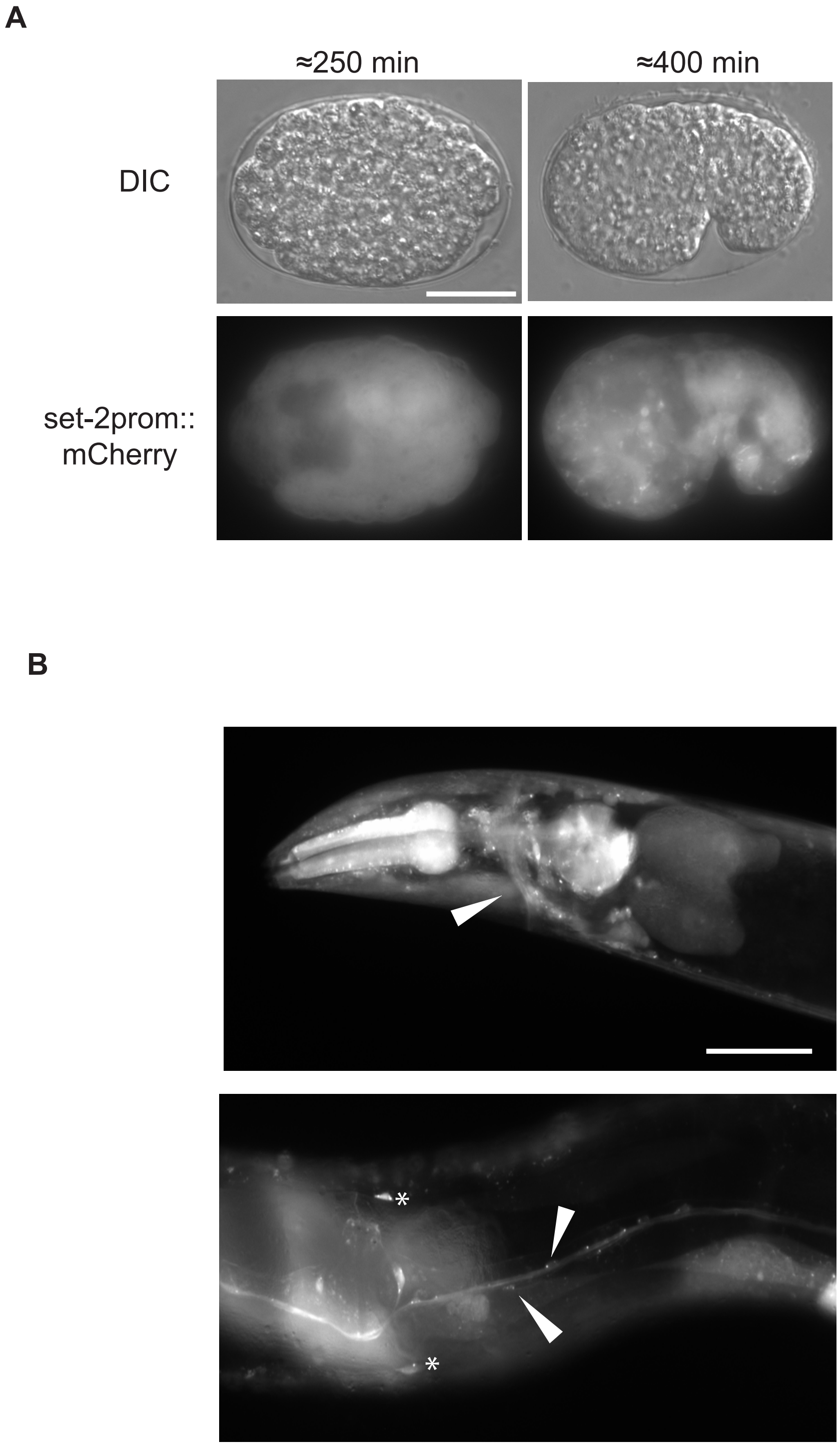

Development • Supplementary information

Figure S3. *set-2* transgene expression. A. Representative DIC (top) and fluorescence (bottom) images of a transgenic embryo expressing a *set-2* transcriptional construct (ZR1053). Ubiquitous expression of *set-2* during embryonic development is evident around the presumptive time of PVQ axon development. Scale bar 20 μm **B.** Representative fluorescence images of one-day adult transgenic animals. Top: Lateral anterior view. *set-2* expression in the pharynx and in the nerve ring (arrowhead). Bottom: Ventral midbody view. *set-2* expression in neurons of the ventral nerve cord (arrowheads). HSNs are indicated by asterisks. Scale bar 30 μm.

Development • Supplementary information

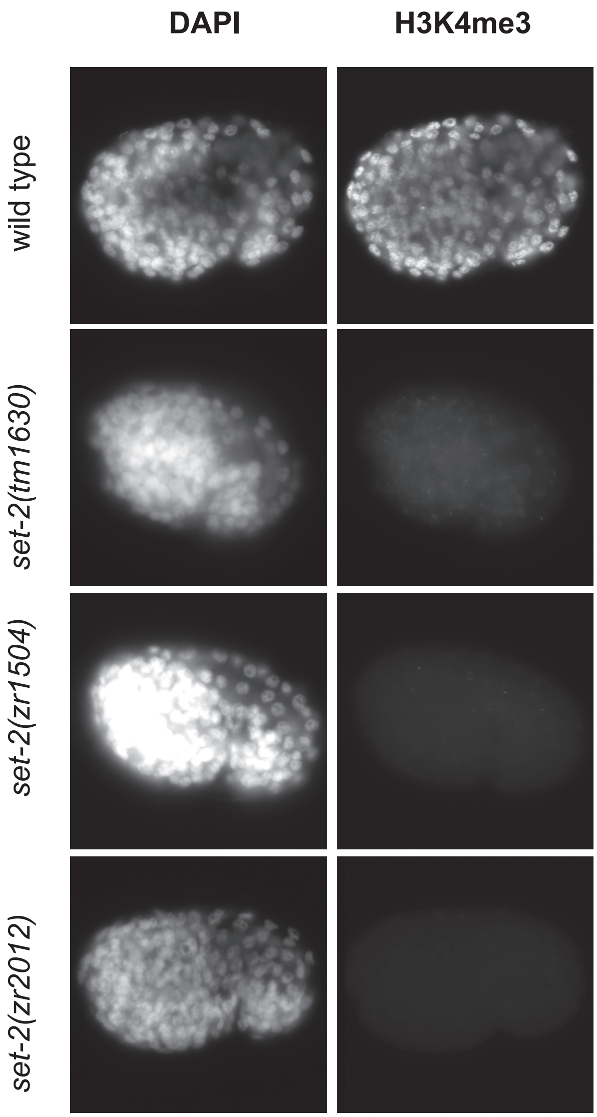

Figure S4. H3K4 levels in *set-2* alleles. Representative images of wild type, *set-2(tm1630)*, *set-2(zr2012)*, *set-2(zr1504)* embryos at comma stage, stained with H3K4me3 antibody. DNA was stained with DAPI.

Development • Supplementary information

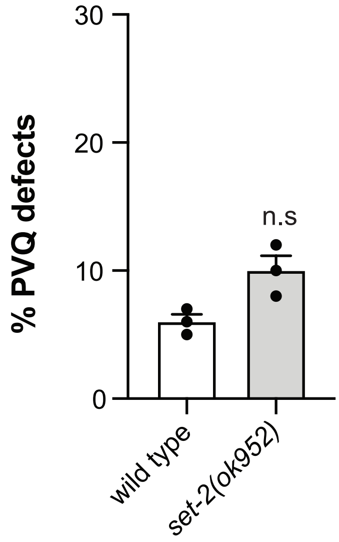

Figure S5. PVQ defects in a weak allele of *set-2*. Quantification of PVQ defects at L4 stage in wild type and *set-2(ok952)* mutants (n>150). Statistical analysis (Student's t-test), n.s, not significant compared to wild type. Black dots represent independent scorings. Error bars represent standard error of mean (SEM).

Development • Supplementary information

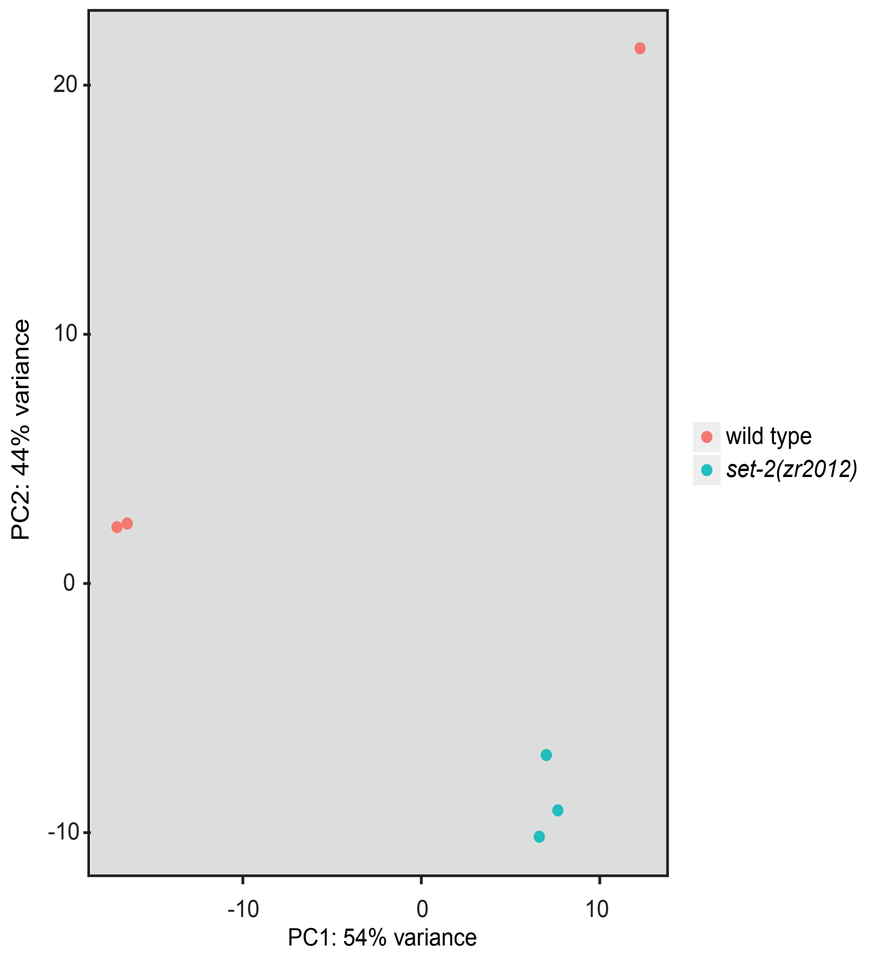

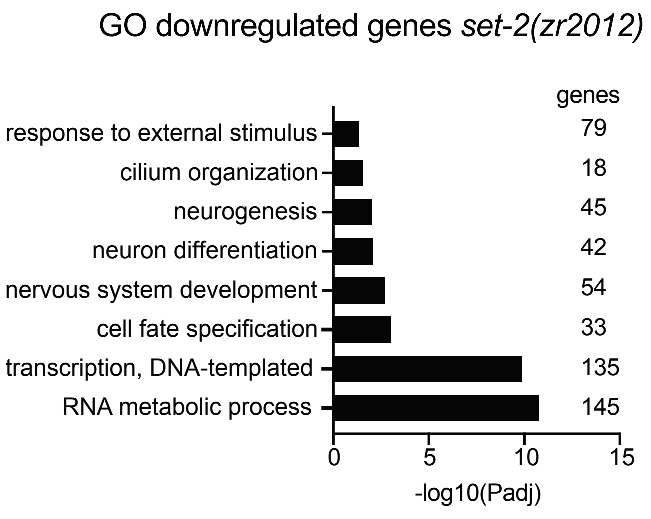

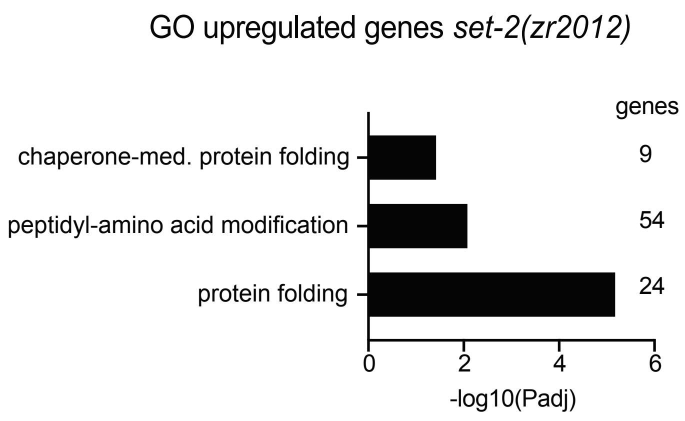

Development • Supplementary information

Figure S6. ***set-2(zr2012)*** **analyses.**

A. Principal component analysis (PCA) plot of wild-type and *set-2(zr2012)* mid-embryos. Each dot represents one sample and each colour a genotype. **B**. Gene ontology analysis of biological processes of down- (left) and up- (right) regulated genes in *set-2(zr2012)* allele by g-Profiler, using adjusted P-values (Bonferroni correction). Selected top scoring categories are presented together with the number of genes identified in each category.

Development • Supplementary information

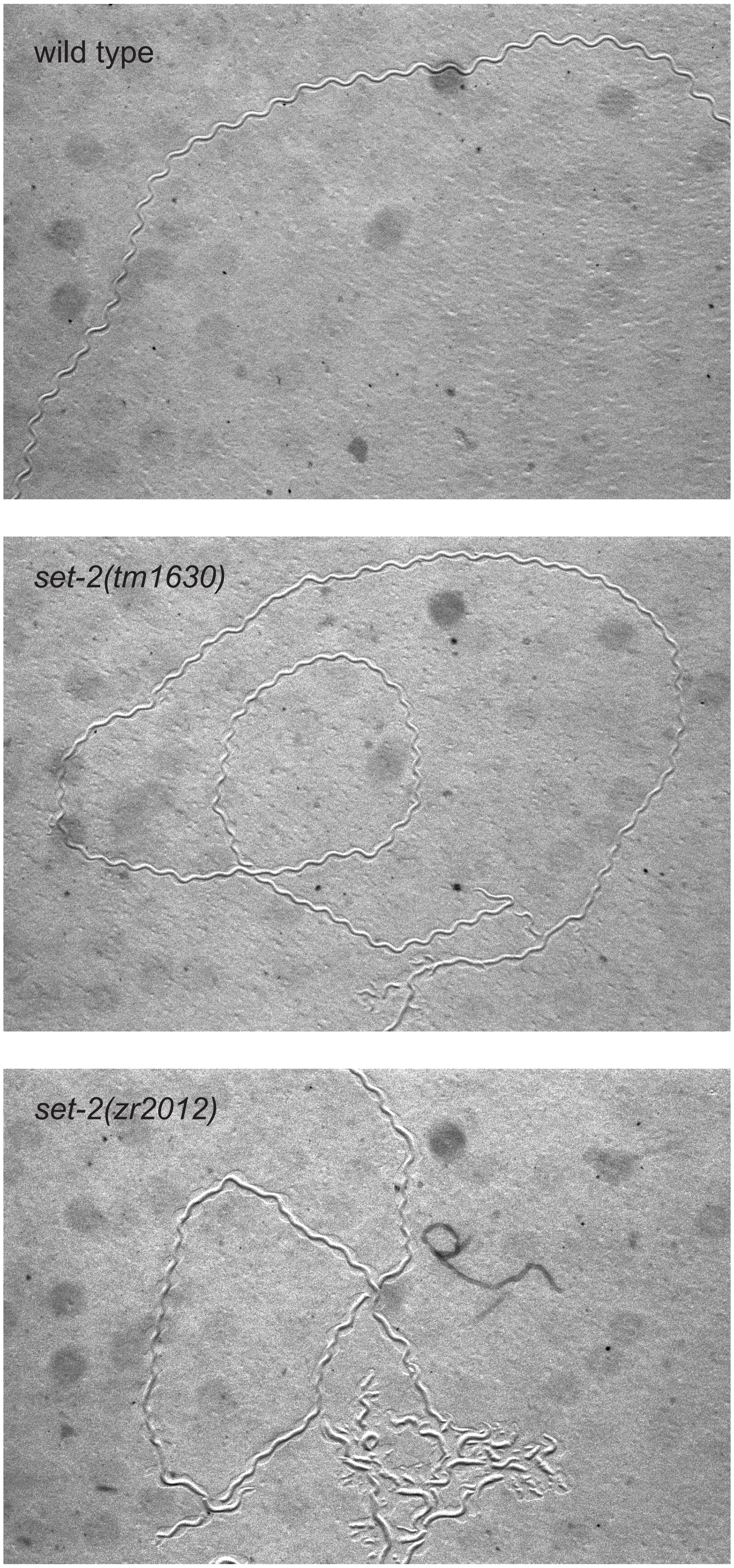

Figure S7. Crawling pattern of *set-2* mutant animals. Representative images of crawling tracks onto an uniform bacterial lawn left by wild type and indicated *set-2* mutant animals.

Table S1. Differentially expressed genes in *set-2* alleles

Click here to Download Table S1

Table S2. List of strains used in this study

Click here to Download Table S2
