# Supplementary material for: Regulators of H3K4 methylation mutated in neurodevelopmental disorders control axon guidance in Caenorhabditis elegans
Source: Development. 2020 Aug 7;147(15):dev190637. doi: 10.1242/dev.190637 (PMC7420840; doi:10.1242/dev.190637)
Supplement: Reviewer comments [file develop-147-190637_review_history.pdf]

Regulators of H3K4 methylation mutated in neurodevelopmental disorders control axon guidance in *Caenorhabditis elegans*

Steffen Abay-Nørgaard, Benedetta Attianese, Laura Boreggio and Anna Elisabetta Salcini
DOI: 10.1242/dev.190637

Editor: Susan Strome

Review timeline

Original submission:	16 March 2020
Editorial decision:	12 May 2020
First revision received:	11 June 2020
Editorial decision:	2 July 2020
Second revision received:	8 July 2020
Accepted:	10 July 2020

Original submission

First decision letter

MS ID#: DEVELOP/2020/190637

MS TITLE: Defective axon guidance links H3K4 deregulation to neurodevelopmental disorders

AUTHORS: Steffen Abay-Noergaard, Benedetta Attianese, Laura Boreggio, and Anna Elisabetta Salcini

I have now received reviews of your manuscript from 3 experts. The reviewers' comments are appended below, or you can access them online: please go to BenchPress and click on the 'Manuscripts with Decisions' queue in the Author Area.

The 3 reviewers are split in their level of enthusiasm for your paper, Reviewer 1 being the most positive and Reviewer 3 having significant concerns about 1) the paper being mainly descriptive, 2) your study not validating your model experimentally, 3) the very high number of genes deregulated in one of the mutants used (6500 out of a total of 20,000 genes), which might be an artifact of poor synchronization, and 4) the parameters used to perform GO analysis (uncorrected p-values are used instead of corrected ones), which are not those normally recommended. Reviewers 1 and 2 raise different points, and all 3 reviewers offer excellent suggestions for improving your study and manuscript. I invite you to consider the reviewers' comments and submit a revised manuscript. Your revised manuscript will be re-reviewed, and acceptance will depend on your satisfactorily addressing the reviewers' concerns. Please note that Development normally permits only one round of 'major revision'.

I am aware that you may currently be unable to access the lab to undertake experimental revisions. If it would be helpful, you are welcome to contact me to discuss your revision in greater detail. If you want to pursue that path, please send me a point-by-point response indicating where you are able to address concerns raised (either experimentally or by changes to the text) and where you will not be able to do so within the normal time frame of a revision. I would then provide further guidance. Please also note that Development is happy to extend revision time frames as necessary.

When you submit a revised manuscript, please clearly HIGHLIGHT all changes made in the revised version. You should avoid using 'Tracked Changes' in Word files as these are lost in PDF conversion. I

© 2020. Published by The Company of Biologists under the terms of the Creative Commons Attribution License (https://creativecommons.org/licenses/by/4.0/).

also request a point-by-point response detailing how you have dealt with the points raised by the reviewers in the 'Response to Reviewers' box. If you do not agree with any of the reviewers' criticisms or suggestions, please explain why.

Reviewer 1

Advance summary and potential significance to field

This work represents a nice set of genetic experiments using C. elegans and uncovered the roles of H3K4 methyl regulators in axon guidance, gene regulation and behavior. The manuscript is clearly written and the quantity and quality of the genetics studies are excellent. The novelty of the work resides in its connection of axon guidance to some of the H3K4 methyl regulators.

Comments for the author

I believe that addressing the following concerns would improve the manuscript.

Major issues.
1. The authors did not find phenotypes in the mutant of set-17 and set-30. Are they really H3K4 methyltransferases? What I believe is that some of their mammalian homologues were first found to be H3K4me transferases; however, later, the biochemical activity was refuted and these were instead shown to methylate other histone lysine residues, such as H3K36. The authors should provide full information about these prior studies. The lack of phenotypes for these two genes appears to be key for the authors to claim the striking correlation with human mutation situations.
2. Fig. 2C rescue experiments. The authors probably should show that these rescue transgenes were indeed expressed in the intended cell types.
3. Based on the similar phenotypes between the promoter deletion mutant and the SET-domain point mutants, the authors claimed that enzymatic activity of set-2 is responsible for the axon guidance phenotype. However, for this claim to be valid, the following two conditions have to be met: 1) the SET-domain mutations must interfere with the catalytic activity. 2) the SET-domain mutations should not interfere with protein stability. The authors mentioned that the mutations are found in human patients and that H3K4me3 levels decrease in the worm mutants. However, these observations do not necessarily satisfy the abovementioned two conditions, because SET-domain proteins are known to have non-catalytic scaffolding functions. From the provided information, a formal possibility can still be that the SET-domain mutations destabilize set-2 without affecting catalytic activity and that loss of set-2 protein led to the H3K4me reduction. The phenotype could still arise due to impairment of the scaffolding function of set-2.
4. Fig 4 A+B and Fig 4C address very different questions; i.e. functional interaction between H3K4me regulators and roles of known axon guidance genes, respectively. I suggest making these into separate figures and/or discussing the results under separate headings.
5. I find that the RNA-seq results were rather superficially described.
Addressing the following points may help:
• What happened to expression of the genes for which genetic interaction with set-2 was tested?
• On the last line of page 9, what is the 2.27 value? Log2 fold change?
• RNA appears to have been extracted from the whole body, so what is the composition of neurons in the whole body? Are these changes occurring within neurons?
• The authors' group appears to have published prior work examining the roles of rbr-2 in axon guidance. Rbr-2 and set-2 genetically interact (Fig. 4A). If the RNA-seq data are available for the rbr-2 mutant, the authors could compare the transcriptomes between the two mutants to see if genetic interaction can be corroborated by the RNA-seq data.

Minor issues.
1. Page 3. This should be Wiedemann-Steiner syndrome.
2. It might be difficult for readers to grasp the roles of axon guidance gene products tested in the study. A diagram depicting the roles of these protein in the axon terminus would help.
3. I am not sure that the title faithfully represents the contents.
Transcriptomic and behavioral changes could well reflect defects in other neuronal functions apart from axon guidance, given the pleiotropic roles of the group of genes tested.

© 2020. Published by The Company of Biologists under the terms of the Creative Commons Attribution License (https://creativecommons.org/licenses/by/4.0/).

Reviewer 2

Advance summary and potential significance to field

In this paper Abay-Nørgaard and colleagues describe the functions of the H3K4 methyltransferase set-2/KMT2F/G. The authors show that mutants in set-2 as well as other genes involved in the regulation of H3K4 methylation display defects in axon guidance of PVQ neurons in C. elegans. These defects are not specific to PVQ, as several other neurons although not all, show defects as well. Transgenic rescue experiments indicate that set-2 functions in neurons, but most likely not cell-autonomously. Point mutant worms that have a mutation analogous to mutations identified in human patients with intellectual disability and autism and are predicted to abrogate enzymatic activity, show the same defects in PVQ neurons and display reduced levels of H3K4 methylation. A genetic analysis indicates that set-2 function in parallel to some known signaling pathways known to be required for PVQ axon guidance, but in the same pathways with others. Surprisingly, genes in the regulators of the actin cytoskeleton are epistatic to loss of function mutations in set-2/KMT2F/G. Not surprisingly, transcriptomic studies reveal large numbers of dysregulated genes in ste-2 mutant and gene ontology studies point to particular dysregulation of gene expression in the nervous system (in addition to other tissues). Finally, the authors demonstrate that in addition to the structural defects in the nervous system of C. elegans, set-2 mutant animals also show deficits in a range of behaviors that can be readily measured in worms including learning and associative memory and locomotion. Overall, this paper is clearly and succinctly written and the claims are supported by the data. While the involvement of epigenetic phenomena in neuronal development mayb not be entirely novel, the set-2 gene has not bee. previously directly shown to be required for neuronal development. A particular strength of the paper is the finding that (a) mutations ste-2 analogous to mutations found in patients with intellectual disability and autism show defects in neuronal patterning and that (b) enzymatic activity of set-2, ie methyl transferase activity is likely required for this process. While intriguing, the genetic findings with the actin regulators are less intuitive and somewhat confusing.
Nonetheless, this paper reports important work that should have an impact on the field.
Pending revisions, this manuscript appears appropriate for Development.

Comments for the author

Points to be addressed:
• The negative data for cell-autonomy, ie the fact that transgenic animals driving expression of set-2 under the sra-6 promoter fail to rescue, is not very strong. There are a number of possible explanations for this negative result, e.g. the promoter may not be active at the right time or at the right levels to achieve rescue. A stronger argument could be made if cell-specific knock out or knock down would not results in no phenotype. Alternatively, the text could be modified accordingly.
• The authors should mention in the text that the embryonic migration of HSN neurons is not compromised in set-2 mutants.
• The authors may also want to report on the larval AVM and PVM migrations, data they should have as well from the reporters the used.
• 'Genetic interactions' is a rather diffuse term, it would be much more useful to describe the genetic interactions briefly that lead to the conclusion that the netrin and slit pathways act in parallel to set-2 whereas the ephrin and semaphorin pathways function in a pathway with set-2
• The authors show data (also fail to mention in the text) that sdn-1 also seems to function in a pathway with set-2. This is a curious result given that sdn-1 has also been shown to function in the Slit pathway (Rhiner et al., 2005), which the authors claim functions in parallel to set-2, based on the double mutant with sax-3. Maybe the authors need to reevaluate their conclusions? Strictly speaking the authors are testing sax-3/Robo and not slt-1 in their assay.
This needs to be cleared up.
• Genetic removal of wsp-1, nck-1 and cdc-42 suppress the set-2 loss of function phenotype. However, the authors show (although fail to mention) that wsp-1 expression levels are reduced in set-2 mutants and nck-1 and cdc-42 transcription levels don't appear to be changed at all in the data set. How do the authors reconcile these findings?
Also, H3K4 methylation is expected to be an activating modification. Hence, how do the authors envision mechanistically, how removal of cdc-42 or nck-1 suppresses the phenotype?

© 2020. Published by The Company of Biologists under the terms of the Creative Commons Attribution License (https://creativecommons.org/licenses/by/4.0/).

The authors should comment on these discrepancies.
• It may be worthwhile to mention the human disease mutation and the effects of the corresponding worm mutation in the discussion section to emphasize this quite important finding that the mutation that is analogous to the patient mutation.
• The F25B3.3 gene has now a name: rgef-1
• Supplementary Figure 1: Title of figure should not claim ‘set-2 expression’ because only expression of a set-2 reporter is shown.
• Given that the authors only backcrossed their set-2 mutants twice they may want to rescue the behavioral defects shown in Fig. 6.

Reviewer 3

Advance summary and potential significance to field

In their manuscript Abay-Nørgaard et al turn to C. elegans to elucidate the role played by the deregulation of H3K4 methylation in several neurodevelopmental disorders. They find that loss-of-function mutations in genes coding for both H3K4 methyltransferases and demethyalses cause axon guidance defects during embryogenesis.
Through transcriptome and GO analyses, the authors identify a set of genes implicated in neuronal functions (including axon guidance), which are deregulated in two different set-2 mutants. They propose a model based on a H3K4 methylation-dependent fine-tuning of gene expression to explain how numerous regulators of H3K4 methylation contribute to the process of axon guidance and to other neuronal functions.
Overall, the authors’ results support the recent hypothesis that axonal guidance defects are a cause of neurodevelopmental disorders and establish an interesting link between the regulation of H3K4 methylation and axonal guidance. However, the mechanistic model proposed is based exclusively on the results of GO analyses, which appear to be problematic for several reasons (see detailed comments below). Furthermore the mechanism presented needs to be substantiated as no evidence is provided that 1) any of the deregulated genes plays a role in axon guidance in a set-2-deficient context, and 2) the transcriptome alteration occurs as a direct consequence of modifications in H3K4 methylation. These are several major points that would need to be addressed before further consideration for publication, but please refer the the list below for further details on how to improve the article and clarifications needed.

Comments for the author

Major comments

1. According to the transcriptomics data 6444 genes are differentially expressed in the set-2 (tm1630) null mutant. This number represents almost a third of the C. elegans genome (20,000 genes). The authors claim that “the majority of eggs was at the comma-stage” but it would be important to provide more details concerning the synchronisation of the embryos used in this experiment. The large number of deregulated genes could simply be an artefact of poor synchronisation between samples. In line with the previous comment it is surprising that less than half the number of genes are differentially expressed in the catalytically dead set-2 (zcr2012) mutant (3053). The authors should comment on this. Also, was PCA analysis performed on the set-2 (zc2012) samples? It would be important to show this along with that of the other strains (figure 5A).

2. When performing their GO term analyses on the up-regulated and down-regulated gene sets identified in the set-2 (tm1630) mutant the authors analyse a number of genes (3288, 3158) higher than the recommend upper limit for the DAVID “Functional Annotation Clustering” (3000). This may increase their false-positive rate. Moreover, for all their GO analyses the authors use the uncorrected p-values to establish significance while it would be more appropriate to use the adjusted p-values (Bonferroni or FDR) to take into account the multiple comparisons problem.

3. It is unclear why the GO analysis is not performed on the deregulated genes identified in the set-2 (zc2012) mutant. The smaller size of this data set would allow to obtain more reliable results.

© 2020. Published by The Company of Biologists under the terms of the Creative Commons Attribution License (https://creativecommons.org/licenses/by/4.0/).

Besides, the catalytically dead mutant is the most appropriate to focus on the catalytic role of SET-2.

4. The GO term analysis carried out on the genes deregulated in both set-2 mutants (1118) identifies several neuronal function groups enlisted in Supplementary Table 1. Unfortunately, the adjusted p-values for the majority of these groups (not shown by the authors) are higher than the threshold of 0.05 normally used to establish significance; for example the "axon guidance" category has a Bonferroni adjusted p-value of 0.45 and a FDR adjusted p-value of 19.8 according to DAVID. Overall, the results of the GO analyses presented are very much influenced by the choice of the p-values when the adjusted values are used, the results no longer support the conclusions drawn by the authors.

5. PVQ defects are detected in mutants lacking H3K4 HMTs (SET-2 and SET-16) as well as in mutants lacking H3K4 demethylases (RBR-2 and SPR-5). This argues against a simple model in which methylation and demethylation play opposing roles in regulating the expression of neuronal programs. The authors instead propose that these antagonistic activities all contribute to the fine-tuning of gene expression required for normal axon guidance. Although appealing, this model needs to be supported by experimental data. For example, the authors could select some of the genes belonging to the axon guidance GO group (common to both set-2 mutants) and confirm that they are indeed down-regulated in highly synchronized set-2 embryos.
This could be done by qRT-PCR and/or in vivo using any of the many available transcriptional reporters for neuronal genes misregulated in these mutants, eg unc-13, unc-6, vab-7. Ideally, once the trancriptomics data are validated few transgenic lines could be generated to check whether overexpression of any of the axonal guidance genes studied reverts the axonal defect phenotype of the set-2 mutants.

6. To support the claim that H3K4 methylation is directly responsible for the gene expression differences observed in the set-2 mutants the authors should at least try performing ChIP-qPCR analysis on the most widely expressed genes on highly synchronized embryos to test whether H3K4me3 is affected at the promoter of a few down-regulated genes. This may be quite challenging given that a single gene is likely to be misregulated in only a handful of cells in the embryo at a given stage, but should be tried. The Salcini Lab has succeed in performing this very same analysis for a previous article (Mariani et al, 2016).

7. In a recent paper (Mukai J, Neuron, 2019) it is shown that schizophrenia-related phenotypes in mice heterozygous for a Setd1a mutation are reversed by repression of the conserved H3K4 demethylase LSD1.
Testing whether loss of the C. elegans homologue lsd-1 suppresses the axon guidance defect of the set-2 mutant could certainly contribute to gain some mechanistic insights into the role played by SET-2 in axon guidance.

8. I understand the authors want to emphasise the role of set-2 in the regulation of axon guidance. However their analysis shows that loss of set-16 affects axon guidance similarly to loss of set-2 (fig. 1B). The authors should comment on this based on the fact that, as observed in C. elegans and mammals, SET-2 and sET-16 may have non-redundant roles and the role of SET-16 seems to be restricted to specific genes.

9. In line with the previous point, the ash-2 and rbbp-5 mutants show a higher % of PVQ defects as compared to both set-2 and set-16 mutants (fig. 1B, is this difference significant?). This might depend on the fact that ASH-2 and RBBP-5 are core components of both COMPASS and MLL-like complexes and may affect the activity of both.

10. In figure 2C neuronal expression of set-2 does not seem to fully rescue the axonal guidance defect of the set-2 mutant. Is the difference between WT and neuronal set-2 significant? If yes, this would suggest that other tissues, yet to be identified, may contribute to the effect of set-2. Alternatively, the partial rescue may depend on the use of an extrachromosomal array; the authors may obtain a full rescue when the transgene is integrated.

© 2020. Published by The Company of Biologists under the terms of the Creative Commons Attribution License (https://creativecommons.org/licenses/by/4.0/).

Minor comments

1. The Salcini lab has previously described a mechanism for how the modification of H3K4 methylation affects axon guidance through activation of wsp-1 expression in rbr-2-deficient animals (Mariani et al, 2016). In a paper cited by the authors (Pocock and Hobert, 2008) up-regulation of vab-1 is responsible for the axon guidance defect caused by hypoxia. How do the authors reconcile these findings with the fact that both wsp-1 and vab-1 are repressed in set-2-deficient animals? Could this be part of an attempt to compensate the defects in actin remodelling?

2. The authors should provide more details about 1) the set-16 mutant used, which is, I assume, a balanced strain, and 2) the protocol used to analyse the set-16 homozygotes.

3. It would be important to add a representative image (to figure 1 or in Supplementary material) showing a crossover of axons in L1 larvae since this is the larval stage used to conclude that SET-2 plays a role in axon guidance during embryogenesis.

4. How did the authors select the H1447K allele? Based on yeast/mammalian studies? Please, explain this in the Results/Methods sections.

5. Page 8, first paragraph, syndecan is enlisted as an “extracellular molecule” but it is instead a transmembrane protein.

6. End on Introduction, the authors write : “In this study, we have directly tested the role of H3K4 methylation in regulating axon guidance by analysing mutant animals lacking all known H3K4 regulators”. This is not correct as, for example, the authors did not include either dpy-30 or cfp-1.

7. On page 7 the authors claim that “SET-2 specifically catalyses the tri-methylation of H3K4”. I think that currently there is not enough evidence to rule out a role of SET-2 in the di-methylation of H3K4.

First revision

<u>Author response to reviewers' comments</u>

We thank the reviewers for questions and constructive criticisms that allowed us to refine and improve our manuscript. Below, in italics, a point-by-point response to the concerns raised by the reviewers.

Point-by-Point response

Reviewer 1 Advance Summary and Potential Significance to Field:
This work represents a nice set of genetic experiments using C. elegans and uncovered the roles of H3K4 methyl regulators in axon guidance, gene regulation, and behavior. **The manuscript is clearly written and the quantity and quality of the genetics studies are excellent. The novelty of the work resides in its connection of axon guidance to some of the H3K4 methyl regulators.**

Reviewer 1 Comments for the Author:
I believe that addressing the following concerns would improve the manuscript.

Major issues.
1. The authors did not find phenotypes in the mutant of set-17 and set-30. Are they really H3K4 methyltransferases? What I believe is that some of their mammalian homologues were first found to be H3K4me transferases; however, later, the biochemical activity was refuted and these were instead shown to methylate other histone lysine residues, such as H3K36. The authors should provide full information about these prior studies. The lack of phenotypes for these two genes appears to be key for the authors to claim the striking correlation with human

© 2020. Published by The Company of Biologists under the terms of the Creative Commons Attribution License (https://creativecommons.org/licenses/by/4.0/).

mutation situations.

We now provide more detailed information regarding set-17 and set-30 and mammalian homologs according to available literature. set-17 shares homology with PRDM7 and PRDM9. According to Blazer et al. 2016, PRDM7 is a H3K4 methyltransferase, while PRDM9 is a methyltransferase for H3K4 (Hayashi et al. 2005) and H3K36 (Eram et al, 2014). set-30 shares homology with SMYD2/KMT3C, shown to methylate both H3K36 (Brown et al, 2006) and H3K4 (Abu-Farha et al, 2008). It should be noted that the catalytic activity of the C. elegans homologs SET-17 and SET-30 as methyltransferase specific for H3K4 has been carefully evaluated in Greer et al, 2014, using both in vitro and in vivo assays. Based on these solid results, we included set-17 and set-30 mutants in our H3K4 modulators screen.

2. Fig. 2C rescue experiments. The authors probably should show that these rescue transgenes were indeed expressed in the intended cell types.

We now provide pictures illustrating the expression of transgenes in Supplementary Figure 1.

3. Based on the similar phenotypes between the promoter deletion mutant and the SET-domain point mutants, the authors claimed that enzymatic activity of set-2 is responsible for the axon guidance phenotype. However, for this claim to be valid, the following two conditions have to be met: 1) the SET-domain mutations must interfere with the catalytic activity. 2) the SET-domain mutations should not interfere with protein stability.

The authors mentioned that the mutations are found in human patients and that H3K4me3 levels decrease in the worm mutants. However, these observations do not necessarily satisfy the abovementioned two conditions, because SET-domain proteins are known to have non-catalytic scaffolding functions. From the provided information, a formal possibility can still be that the SET-domain mutations destabilize set-2 without affecting catalytic activity and that loss of set-2 protein led to the H3K4me reduction. The phenotype could still arise due to impairment of the scaffolding function of set-2.

We fully agree with the reviewer. While widely used to establish the relevance of the catalytic activity of an enzyme, point mutants may result in protein instability, therefore we will moderate our conclusion and include this possibility in the text. However, in this context, it should also be noted that the set-2(ok952) allele conserves the ability of methylate H3K4 and does not show the axon defects, corroborating the relevance of the catalytic activity in axon guidance process.

4. Fig 4 A+B and Fig 4C address very different questions; i.e. functional interaction between H3K4me regulators and roles of known axon guidance genes, respectively. I suggest making these into separate figures and/or discussing the results under separate headings.

We now discuss the results illustrated in Figure 4 in separate headings.

5. I find that the RNA-seq results were rather superficially described. Addressing the following points may help:
•What happened to expression of the genes for which genetic interaction with set-2 was tested?

By RNA sequencing, axon guidance genes tested in genetic approaches are downregulated. We now report this observation in the result section.

•On the last line of page 9, what is the 2.27 value? Log2 fold change?

2.27 is the median log2 fold change for upregulated genes in set-2 mutant. We now provide a better description in the text.

•RNA appears to have been extracted from the whole body, so what is the composition of neurons in the whole body? Are these changes occurring within neurons?

© 2020. Published by The Company of Biologists under the terms of the Creative Commons Attribution License (https://creativecommons.org/licenses/by/4.0/).

The RNA analysis has been performed in late whole embryos. We cannot and don't assume that the changes are specific for neuronal cells.

•The authors' group appears to have published prior work examining the roles of rbr-2 in axon guidance. Rbr-2 and set-2 genetically interact (Fig. 4A). If the RNA-seq data are available for the rbr-2 mutant, the authors could compare the transcriptomes between the two mutants to see if genetic interaction can be corroborated by the RNA-seq data.

Unfortunately, the RNA seq data on rbr-2 mutant animals at embryo stage are not available.

Minor issues.
1. Page 3. This should be Wiedemann-Steiner syndrome.

We corrected the mistake in the revised manuscript.

2. It might be difficult for readers to grasp the roles of axon guidance gene products tested in the study. A diagram depicting the roles of these protein in the axon terminus would help.

We now propose a diagram depicting the hypothetical roles of these protein and highlighting our main findings in the new figure 7.

3. I am not sure that the title faithfully represents the contents. Transcriptomic and behavioral changes could well reflect defects in other neuronal functions apart from axon guidance, given the pleiotropic roles of the group of genes tested.

We changed the title in "Defective axon guidance links H3K4 regulators to neurodevelopmental disorders" (instead of "Defective axon guidance links H3K4 deregulation to neurodevelopmental disorders"). We think that the new title still highlights our main finding, that all genes found mutated in neurodevelopmental diseases are involved in the process of axon guidance in C. elegans, without overinterpreting the results.

Reviewer 2 Advance Summary and Potential Significance to Field:
In this paper Abay-Nørgaard and colleagues describe the functions of the H3K4 methyltransferase set-2/KMT2F/G. The authors show that mutants in set-2 as well as other genes involved in the regulation of H3K4 methylation display defects in axon guidance of PVQ neurons in C. elegans. These defects are not specific to PVQ, as several other neurons, although not all, show defects as well. Transgenic rescue experiments indicate that set-2 functions in neurons, but most likely not cell-autonomously. Point mutant worms that have a mutation analogous to mutations identified in human patients with intellectual disability and autism and are predicted to abrogate enzymatic activity, show the same defects in PVQ neurons and display reduced levels of H3K4 methylation. A genetic analysis indicates that set-2 function in parallel to some known signaling pathways known to be required for PVQ axon guidance, but in the same pathways with others. Surprisingly, genes in the regulators of the actin cytoskeleton are epistatic to loss of function mutations in set- 2/KMT2F/G. Not surprisingly, transcriptomic studies reveal large numbers of dysregulated genes in ste-2 mutant and gene ontology studies point to particular dysregulation of gene expression in the nervous system (in addition to other tissues). Finally, the authors demonstrate that in addition to the structural defects in the nervous system of C. elegans, set-2 mutant animals also show deficits in a range of behaviors that can be readily measured in worms, including learning and associative memory and locomotion. **Overall, this paper is clearly and succinctly written and the claims are supported by the data. While the involvement of epigenetic phenomena in neuronal development mayb not be entirely novel, the set-2 gene has not bee. previously directly shown to be required for neuronal development. A particular strength of the paper is the finding that (a) mutations ste-2 analogous to mutations found in patients with intellectual disability and autism show defects in neuronal patterning and that (b) enzymatic activity of set-2, ie methyl transferase activity is likely required for this process.** While intriguing, the genetic findings with the actin regulators are less intuitive and somewhat confusing. **Nonetheless, this paper reports important work that should have an impact on the field.** Pending revisions, this manuscript appears appropriate for Development.

© 2020. Published by The Company of Biologists under the terms of the Creative Commons Attribution License (https://creativecommons.org/licenses/by/4.0/).

Reviewer 2 Comments for the Author:
Points to be addressed:

•The negative data for cell-autonomy, ie the fact that transgenic animals driving expression of set-2 under the sra-6 promoter fail to rescue, is not very strong. There are a number of possible explanations for this negative result, e.g. the promoter may not be active at the right time or at the right levels to achieve rescue. A stronger argument could be made, if cell-specific knock out or knock down would not results in no phenotype. Alternatively, the text could be modified accordingly.

We agree with the reviewer and include this possibility in the revised manuscript.

•The authors should mention in the text that the embryonic migration of HSN neurons is not compromised in set-2 mutants.

Done.

•The authors may also want to report on the larval AVM and PVM migrations, data they should have as well from the reporters the used.

We now provide these data. We inserted the results in Table 2 and mention them in the text.

•'Genetic interactions' is a rather diffuse term, it would be much more useful to describe the genetic interactions briefly that lead to the conclusion that the netrin and slit pathways act in parallel to set-2 whereas the ephrin and semaphorin pathways function in a pathway with set-2

We provide now a more detailed description of the genetic interactions in the main text.

•The authors show data (also fail to mention in the text) that sdn-1 also seems to function in a pathway with set-2. This is a curious result given that sdn-1 has also been shown to function in the Slit pathway (Rhiner et al., 2005), which the authors claim functions in parallel to set-2, based on the double mutant with sax-3. Maybe the authors need to reevaluate their conclusions? Strictly speaking the authors are testing sax-3/Robo and not slt-1 in their assay. This needs to be cleared up.

The genetic interaction between sdn-1 and slt-1/sax-3/ROBO pathway is based on sdn- 1;slt-1 double mutant analysis (Rheiner et al, 2005). In our genetic texts, we used instead sax-3;set-2 double mutant. The discrepancy, correctly pointed out by the reviewer, could be related to the evidence that sax-3 has slt-1-independent roles, as suggested in Hao et al, 2001, based on the fact that the PVQ defects observed in sax-3 mutant are more pronounced compared to the ones presented by slt-1 mutant. Therefore, our genetic interaction tests may suggest that set-2 acts in parallel to a slt-1-independent sax-3 pathway. We thank the reviewer for highlighting this incongruity. We now report that we test sax-3 (and not slt-1) pathway.

•Genetic removal of wsp-1, nck-1 and cdc-42 suppress the set-2 loss of function phenotype. However, the authors show (although fail to mention) that wsp-1 expression levels are reduced in set-2 mutants and nck-1 and cdc-42 transcription levels don't appear to be changed at all in the data set. How do the authors reconcile these findings? Also, H3K4 methylation is expected to be an activating modification. Hence, how do the authors envision mechanistically, how removal of cdc-42 or nck-1 suppresses the phenotype? The authors should comment on these discrepancies.

We thank the reviewer for this question that allowed us to better clarify this important point. Actin remodeling is complex and controlled at multiple level. We think that a plausible explanation is that actin reorganization is regulated not only by the expression level of wsp- 1 but also by WSP-1 activation, that is depending from post-translational modifications such as phosphorylation and from its localization in the cells (cytoplasm/plasma membrane- bound) mediated for example by cdc-42. It is therefore likely that the expression level of wsp-1, nck-1 and cdc-42 is not the only parameter to consider. As ablation of wsp-1 does not affect axon

© 2020. Published by The Company of Biologists under the terms of the Creative Commons Attribution License (https://creativecommons.org/licenses/by/4.0/).

guidance, it is likely that the downregulation of wsp-1 transcription observed in set-2 mutants is the result of a compensatory effect balancing the increased activity. We highlight this hypothesis in discussion and mention the downregulation of wsp-1 levels.

•It may be worthwhile to mention the human disease mutation and the effects of the corresponding worm mutation in the discussion section to emphasize this quite important finding that the mutation that is analogous to the patient mutation.

We now stress this point further in discussion.

•The F25B3.3 gene has now a name: rgef-1

Done.

•Supplementary Figure 1: Title of figure should not claim 'set-2 expression' because only expression of a set-2 reporter is shown.

Done.

•Given that the authors only backcrossed their set-2 mutants twice, they may want to rescue the behavioral defects shown in Fig. 6.

We candidly used in Material and Methods the expression "set-2 alleles were backcrossed at least two times". However, the set-2(tm1630) allele, used in all experiments described in the manuscript, has been backcrossed 6 times before the analyses. The set-2(zr2012) allele has been backcrossed 2 times. We clarify this point in the Material and Methods section.
It should be also noted that the behavioral tests are all performed using two set-2 alleles, thus providing strong evidence that the phenotypes observed are associate to loss of set-2 and not to unrelated mutations.

Reviewer 3 Advance Summary and Potential Significance to Field:
In their manuscript Abay-Nørgaard et al turn to C. elegans to elucidate the role played by the deregulation of methylation in several neurodevelopmental disorders. They find that loss-of-function mutations in genes coding for both H3K4 methyltransferases and demethyalses cause axon guidance defects during embryogenesis.
Through transcriptome and GO analyses, the authors identify a set of genes implicated in neuronal functions (including axon guidance), which are deregulated in two different set-2 mutants. They propose a model based on a H3K4 methylation-dependent fine-tuning of gene expression to explain how numerous regulators of H3K4 methylation contribute to the process of axon guidance and to other neuronal functions. Overall, the authors' results support the recent hypothesis that axonal guidance defects are a cause of neurodevelopmental disorders **and establish an interesting link between the regulation of H3K4 methylation and axonal guidance.**
However, the mechanistic model proposed is based exclusively on the results of GO analyses, which appear to be problematic for several reasons (see detailed comments below). Furthermore, the mechanism presented needs to be substantiated as no evidence is provided that 1) any of the deregulated genes plays a role in axon guidance in a set-2-deficient context, and 2) the transcriptome alteration occurs as a direct consequence of modifications in H3K4 methylation. These are several major points that would need to be addressed before further consideration for publication, but please refer the the list below for further details on how to improve the article and clarifications needed.

Reviewer 3 Comments for the Author:

Major comments

1. According to the transcriptomics data 6444 genes are differentially expressed in the set-2 (tm1630) null mutant. This number represents almost a third of the C. elegans genome (20,000 genes). The authors claim that "the majority of eggs was at the comma-stage" but it would be important to provide more details concerning the synchronisation of the embryos used in this

© 2020. Published by The Company of Biologists under the terms of the Creative Commons Attribution License (https://creativecommons.org/licenses/by/4.0/).

experiment. The large number of deregulated genes could simply be an artefact of poor synchronisation between samples. In line with the previous comment it is surprising that less than half the number of genes are differentially expressed in the catalytically dead set-2 (zcr2012) mutant (3053). The authors should comment on this.

We recognize that a perfect synchronization of embryos is not feasible and therefore some of the difference observed between the two set-2 alleles are likely depending on this technical issue. We now provide details of the sample preparation (hermaphrodites were double bleached to reach a better synchronization, wild-type and set-2 samples have been prepared at the same time and >80% of the embryos were at comma stage). We can also envision another possible explanation for these differences, that might relate to non- catalytic function of set-2 (e.g. assembly of the COMPASS Complex) in transcription that, however, require further analysis before to be considered.

Also, was PCA analysis performed on the set-2 (zc2012) samples? It would be important to show this along with that of the other strains (figure 5A).

We now show this information in Supplementary Figure 5.

2. When performing their GO term analyses on the up-regulated and down-regulated gene sets identified in the set-2 (tm1630) mutant the authors analyse a number of genes (3288, 3158) higher than the recommend upper limit for the DAVID “Functional Annotation Clustering” (3000). This may increase their false-positive rate. Moreover, for all their GO analyses the authors use the uncorrected p-values to establish significance while it would be more appropriate to use the adjusted p-values (Bonferroni or FDR) to take into account the multiple comparisons problem.

We thank the reviewer to point out this problem. We repeated the GO analyses using g-Profiler and adjusted P value (by Bonferroni) to identify enriched categories. We obtained very similar results. We now present the data using adjusted P-values in the revised manuscript (Figure 5). We think that, by reporting these new analyses in the revised manuscript, we addressed the most critical point raised by the reviewer, confirming the quality of the data and the absence of over-interpretation.

3. It is unclear why the GO analysis is not performed on the deregulated genes identified in the set-2 (zc2012) mutant. The smaller size of this data set would allow to obtain more reliable results. Besides, the catalytically dead mutant is the most appropriate to focus on the catalytic role of SET-2.

As in this manuscript we are not focusing specifically on the catalytic activity of SET-2, we think it is more important to present the analysis of the null mutant (tm1630), publicly available from CGC and used by other laboratories.

4. The GO term analysis carried out on the genes deregulated in both set-2 mutants (1118) identifies several neuronal function groups enlisted in Supplementary Table 1. Unfortunately, the adjusted p-values for the majority of these groups (not shown by the authors) are higher than the threshold of 0.05 normally used to establish significance; for example the “axon guidance” category has a Bonferroni adjusted p-value of 0.45 and a FDR adjusted p-value of 19.8 according to DAVID.
Overall, the results of the GO analyses presented are very much influenced by the choice of the p-values, when the adjusted values are used, the results no longer support the conclusions drawn by the authors.

We repeated the analysis of this set of genes using G profiler with adjusted P-value by Bonferroni and obtained very similar results. We report this new analysis in the revised manuscript (Figure 5 and Supplementary table 1).

5. PVQ defects are detected in mutants lacking H3K4 HMTs (SET-2 and SET-16) as well as in mutants lacking H3K4 demethylases (RBR-2 and SPR-5). This argues against a simple model in which methylation and demethylation play opposing roles in regulating the expression of

© 2020. Published by The Company of Biologists under the terms of the Creative Commons Attribution License (https://creativecommons.org/licenses/by/4.0/).

neuronal programs. The authors instead propose that these antagonistic activities all contribute to the fine-tuning of gene expression required for normal axon guidance. Although appealing, this model needs to be supported by experimental data.

We fully agree with the reviewer that a simple model cannot fully explain how both methyltransferases and demethylases act to regulate axon guidance, especially considering that these enzymes are contributing to the deposition and the removal of H3K4me1/2/3, at different regulatory regions of the genome. However, our genetic approaches highlight a specific balancing effect of rbr-2 mutation in the context of set-2 genetic background, providing experimental data to our hypothesis.

For example, the authors could select some of the genes belonging to the axon guidance GO group (common to both set-2 mutants) and confirm that they are indeed down- regulated in highly synchronized set-2 embryos. This could be done by qRT-PCR and/or in vivo using any of the many available transcriptional reporters for neuronal genes misregulated in these mutants, eg unc-13, unc-6, vab-7. Ideally, once the trancriptomics data are validated few transgenic lines could be generated to check whether overexpression of any of the axonal guidance genes studied reverts the axonal defect phenotype of the set-2 mutants.

As the axonal phenotype observed in set-2 mutants is likely (based on the RNA sequencing and genetic interactions) the results of a deregulation of many loci, it is unlikely that the overexpression of single gene will be sufficient to restore a wild-type condition. Moreover, it will be very difficult to reproduce the proper temporal/spatial expression pattern using transgenes. Overall, the approached suggested might lead to inconclusive results that, in any case, will not change the message of our manuscript.

6. To support the claim that H3K4 methylation is directly responsible for the gene expression differences observed in the set-2 mutants the authors should at least try performing ChIP-qPCR analysis on the most widely expressed genes on highly synchronized embryos to test whether H3K4me3 is affected at the promoter of a few down- regulated genes. This may be quite challenging given that a single gene is likely to be misregulated in only a handful of cells in the embryo at a given stage, but should be tried. The Salcini Lab has succeed in performing this very same analysis for a previous article (Mariani et al, 2016).

We fully agree with the point raised by the reviewer. That H3K4 methylation has an instructive or a correlative function in transcription is one of the most outstanding question in the field of epigenetics. To proper show that SET-2 activity correlates with deposition of H3K4me3 and active gene transcription, extensive biochemical approaches are required, including Chromatin Immunoprecipitation/sequencing (or qPCR) for H3K4me3 and for SET- 2 to be compared to transcriptome profiles. We think that this extensive approach is out of the scope of the submitted manuscript.

7. In a recent paper (Mukai J, Neuron, 2019) it is shown that schizophrenia-related phenotypes in mice heterozygous for a Setd1a mutation are reversed by repression of the conserved H3K4 demethylase LSD1. Testing whether loss of the C. elegans homologue lsd-1 suppresses the axon guidance defect of the set-2 mutant could certainly contribute to gain some mechanistic insights into the role played by SET-2 in axon guidance.

We tested and reported the effect of spr-5 loss (Fig. 4A), that is considered the ortholog of LSD1 in worms (Katz et al, 2010). The suppression observed in mammals seems to do not occur in C. elegans, as the double mutant spr-5;set-2 shows axonal defects.

8. I understand the authors want to emphasise the role of set-2 in the regulation of axon guidance. However, their analysis shows that loss of set-16 affects axon guidance similarly to loss of set-2 (fig. 1B). The authors should comment on this based on the fact that, as observed in C. elegans and mammals, SET-2 and sET-16 may have non-redundant roles and the role of SET-16 seems to be restricted to specific genes.

We now comment on this possibility in discussion.

© 2020. Published by The Company of Biologists under the terms of the Creative Commons Attribution License (https://creativecommons.org/licenses/by/4.0/).

9. In line with the previous point, the ash-2 and rbbp-5 mutants show a higher % of PVQ defects as compared to both set-2 and set-16 mutants (fig. 1B, is this difference significant?). This might depend on the fact that ASH-2 and RBBP-5 are core components of both COMPASS and MLL-like complexes and may affect the activity of both.

This is an interesting hypothesis. The level of defects observed in set-2 is significantly different from the ones observed in ash-2 (P=0.0024) and rbbp-5 (P=0.0010), but the level of set-16 defects is not (P=0.126 and P=0.0582, respectively). Based on these results, we cannot assume that the higher level of defects observed in ash-2 and rbbp-5 depends on the fact that loss of these core components compromises both the COMPASS and the MLL-like complex. To approach properly this possibility, direct targets of set-2 and set-16 should be identified. Unfortunately, the reagents required for this experiment are at the moment not available.

10. In figure 2C neuronal expression of set-2 does not seem to fully rescue the axonal guidance defect of the set-2 mutant. Is the difference between WT and neuronal set-2 significant? If yes, this would suggest that other tissues, yet to be identified, may contribute to the effect of set-2. Alternatively, the partial rescue may depend on the use of an extrachromosomal array; the authors may obtain a full rescue when the transgene is integrated.

The difference between WT and the transgenic line showing the lowest percent of defect is not significant (p=0.166), suggesting that re-expression of set-2 in the neuronal tissue is sufficient to achieve correct axon guidance.

Minor comments

1. The Salcini lab has previously described a mechanism for how the modification of H3K4 methylation affects axon guidance through activation of wsp-1 expression in rbr-2-deficient animals (Mariani et al, 2016). In a paper cited by the authors (Pocock and Hobert, 2008) up-regulation of vab-1 is responsible for the axon guidance defect caused by hypoxia. How do the authors reconcile these findings with the fact that both wsp-1 and vab-1 are repressed in set-2-deficient animals? Could this be part of an attempt to compensate the defects in actin remodelling?

As stated before (see responses to reviewer 2), deregulation of gene expression is likely not the only component responsible for axon guidance defects. Localization, secretion, trafficking and activation of several molecules are likely also very important in this process. Clearly, more work, including the identification of direct targets, is required to understand how these molecules and pathways integrate to coordinate the axon guidance process. It is expected that compensatory mechanisms take place when multiple mechanisms are involved, as correctly suggested by the reviewer.

2. The authors should provide more details about 1) the set-16 mutant used, which is, I assume, a balanced strain, and 2) the protocol used to analyse the set-16 homozygotes.

We included the information in Material and Methods.

3. It would be important to add a representative image (to figure 1 or in Supplementary material) showing a crossover of axons in L1 larvae since this is the larval stage used to conclude that SET-2 plays a role in axon guidance during embryogenesis.

We now show a representative image of a defect occurring at L1 stage in supplementary Fig. 2.

4. How did the authors select the H1447K allele? Based on yeast/mammalian studies? Please, explain this in the Results/Methods sections.

The selection was based on the following criteria: 1) conserved residue from yeast to human, 2) outside the interaction surface with other components of the MLL-complex, based on Shinsky et al, 2014 , 3) conservative substitution (H to K). We included this information in

© 2020. Published by The Company of Biologists under the terms of the Creative Commons Attribution License (https://creativecommons.org/licenses/by/4.0/).

Material and Methods.

5. Page 8, first paragraph, syndecan is enlisted as an “extracellular molecule” but it is instead a transmembrane protein.

We corrected this mistake

6. End on Introduction, the authors write: “In this study, we have directly tested the role of H3K4 methylation in regulating axon guidance by analysing mutant animals lacking all known H3K4 regulators”. This is not correct as, for example, the authors did not include either dpy-30 or cfp-1.

We agree, and we changed this statement with “the majority of the known H3K4 regulators” in the text.

7. On page 7 the authors claim that “SET-2 specifically catalyses the tri-methylation of H3K4”. I think that currently there is not enough evidence to rule out a role of SET-2 in the di-methylation of H3K4.

We agree with the reviewer and we rephrased in: SET-2 mainly catalyzes the tri- methylation of H3K4.

Second decision letter

MS ID#: DEVELOP/2020/190637

MS TITLE: Defective axon guidance links H3K4 regulators to neurodevelopmental disorders

AUTHORS: Steffen Abay-Noergaard, Benedetta Attianese, Laura Boreggio, and Anna Elisabetta Salcini

I have now received reviews of your manuscript from the original 3 reviewers. Their comments are appended below, or you can access them online: please go to BenchPress and click on the 'Manuscripts with Decisions' queue in the Author Area.

The 3 reviewers are for the most part satisfied with your revised manuscript but have a few lingering concerns. Notably, Reviewer 1 does not think that the deletion mutant strengthens your argument, and does not approve of your new title, since many-most of the changes in gene expression and behavior may be unrelated to axon guidance; and Reviewer 3 requests further work on your presentation of GO analysis, which I agree wtth. Please consider the reviewers’ comments and submit a revised manuscript.A possible new title for you to consider is: "Deregulation of H3K4 methylation causes defects in axon guidance in C. elegans", which is a simple statement of your most impactful finding.

When you submit a revised manuscript, please clearly HIGHLIGHT all changes made in the revised version. You should avoid using 'Tracked Changes' in Word files as these are lost in PDF conversion. I also request a point-by-point response detailing how you have dealt with the points raised by the reviewers in the 'Response to Reviewers' box. If you do not agree with any of the reviewers’ criticisms or suggestions, please explain why.

Reviewer 1

Advance summary and potential significance to field

The authors adequately addressed many of my concerns.

© 2020. Published by The Company of Biologists under the terms of the Creative Commons Attribution License (https://creativecommons.org/licenses/by/4.0/).

Comments for the author

Two concerns remained

1. With regard to the enzymatic activity, the deletion mutant the authors mentioned has an even higher probability of being degraded than point mutants.
Hence, the deletion mutant does not necessarily strengthen the argument. The authors can cite papers describing the unaffected stability of these mutant proteins.

2. I don't think that the change of title addresses my original concern. Most changes in gene expression and behavior can be unrelated to axon guidance.

Reviewer 2

Advance summary and potential significance to field

as before

Comments for the author

In my opinion, the authors have answered the reviewers' comments satisfactorily – certainly mine. The manuscript is appropriate for publication in Development.

Minor typos:
• P.13:: "help to clarify the specific mechanism underlining the genetic interations observed" should read: "help to clarify the specific mechanism underlYing the genetic interaCtions observed
• P.15: I still found one "F25B3.3", should be replaced by rgef-1
• Legend to Suppl Fig. 2: correct spelling "Rapresentative" to "rEpresentative"

Reviewer 3

Advance summary and potential significance to field

Please, see my first review.

Comments for the author

Abay-Norgaard et al made a significant effort to address the criticism of all the reviewers and, overall, their article has been very much improved.

Concerning my review and, more specifically, the lack of experimental evidence supporting the contribution of H3K4 methylation to axonal guidance through the deregulation of neuronal gene expression, I was disappointed to see that no attempt was made to address the point. However, I understand that the experiments proposed may require several months of work and, in same cases, not be conclusive (points 5 and 6). Given the general interest in understanding the functions of H3K4 methylation in gene expression regulation, the authors may then consider follow-up studies aimed at substantiating the model proposed here.

Minor comments

1. The authors repeated the GO analysis using a different software (g-Profiler). What is the upper limit of genes recommended for this software? I am still concerned about the false-positive rate.

2. I still think that the GO analysis for the set-2 (zr2012) mutant should be included at least in the Supplementary information. A comparison between the tm1630 and zr2012 GO analyses could be informative and provide hints on which functions are directly affected by the methylation of H3K4 and which ones are not.

© 2020. Published by The Company of Biologists under the terms of the Creative Commons Attribution License (https://creativecommons.org/licenses/by/4.0/).

3. I think the commonly deregulated genes (Table S1) should be divided in UP and DOWN to allow the reader to know which ones are consistently up- or down-regulated in both mutants. The genes not consistently deregulated should be indicated and excluded them from the GO analysis.

Second revision

Author response to reviewers' comments

Point-by-point response to reviewers

Reviewer 1 Advance summary and potential significance to field...
The authors adequately addressed many of my concerns.

Reviewer 1 Comments for the author...
Two concerns remained

1. With regard to the enzymatic activity, the deletion mutant the authors mentioned has an even higher probability of being degraded than point mutants. Hence, the deletion mutant does not necessarily strengthen the argument.

Response: in our opinion, the evidence that the set-2(ok952) allele has almost intact level of H3K4 methylation (Xiao et al, 2011) and does not show phenotypes reported for the set-2 null allele (Herbette et al, 2017) or acts as a hypomorph allele (e.g. Antonacci et al, 2015), suggests that set-2(ok952) produces a stable/functional protein. We think that this evidence is a valid argument to support our hypothesis that the catalytic activity of SET-2 is important for the axonal defects.

The authors can cite papers describing the unaffected stability of these mutant proteins.

Response: we now insert two references (Dorighi et al, 2017, Rickels et al, 2017) describing point mutants in SET domain that do not affect the stability of the protein.

2. I don't think that the change of title addresses my original concern. Most changes in gene expression and behavior can be unrelated to axon guidance.

Response: we propose a new title: Regulators of H3K4 methylation mutated in Neurodevelopmental Disorders control Axon Guidance in C. elegans.

Reviewer 2 Advance summary and potential significance to field...
as before

Reviewer 2 Comments for the author...
In my opinion, the authors have answered the reviewers' comments satisfactorily – certainly mine. The manuscript is appropriate for publication in Development.

Minor typos:
• P.13:: "help to clarify the specific mechanism underlining the genetic interations observed" should read: "help to clarify the specific mechanism underlYing the genetic interaCtions observed
• P.15: I still found one "F25B3.3", should be replaced by rgef-1
• Legend to Suppl Fig. 2: correct spelling "Rapresentative" to "rEpresentative"

Response: we thank the reviewer for positive comments. We corrected the typos indicated.

Reviewer 3 Advance summary and potential significance to field...
Please, see my first review.

© 2020. Published by The Company of Biologists under the terms of the Creative Commons Attribution License (https://creativecommons.org/licenses/by/4.0/).

Reviewer 3 Comments for the author...
Abay-Norgaard et al made a significant effort to address the criticism of all the reviewers and, overall, their article has been very much improved.
Concerning my review and, more specifically, the lack of experimental evidence supporting the contribution of H3K4 methylation to axonal guidance through the deregulation of neuronal gene expression, I was disappointed to see that no attempt was made to address the point. However, I understand that the experiments proposed may require several months of work and, in same cases, not be conclusive (points 5 and 6). Given the general interest in understanding the functions of H3K4 methylation in gene expression regulation, the authors may then consider follow-up studies aimed at substantiating the model proposed here.

Response: the interest in establishing a causative connection between H3K4 methylation and gene expression is very high in the field and we are currently approaching this point using suitable genetic and biochemical setups.

Minor comments

1. The authors repeated the GO analysis using a different software (g-Profiler). What is the upper limit of genes recommended for this software? I am still concerned about the false-positive rate.

Response: g-Profiler has no limitation in the number of genes in the query. To avoid false-positive, we used stringent conditions (FDR<005 for DE genes and Bonferroni Correction for the GO analysis).

2. I still think that the GO analysis for the set-2 (zr2012) mutant should be included at least in the Supplementary information. A comparison between the tm1630 and zr2012 GO analyses could be informative and provide hints on which functions are directly affected by the methylation of H3K4 and which ones are not.

Response: we included the GO analysis for the zr2012 allele in Fig. S6.
The categories among the down-regulated genes are similar to the ones identified for the tm1630 allele, with a strong enrichment for neuronal categories. It is of note that many categories enriched in upregulated genes in the tm1630 allele are not found in the analysis of the zr2012 allele, suggesting that upregulated genes may be indirect or catalytic-independent targets. More analyses are required for testing this hypothesis and, as stated above, we are addressing this point using appropriated experimental conditions.

3. I think the commonly deregulated genes (Table S1) should be divided in UP and DOWN to allow the reader to know which ones are consistently up- or down-regulated in both mutants. The genes not consistently deregulated should be indicated and excluded them from the GO analysis.

Response: in table S1, the list of common set-2(tm1630) and set-2(zr2012) DE genes is now complemented by 2 lists of consistently up- and consistently down-regulated genes in the two alleles. We repeated the GO analysis on genes that are consistently down-regulated and inserted the results in Table S1. This analysis highlights an enrichment of neuronal categories among the down-regulated genes, confirming the previous results.

Third decision letter

MS ID#: DEVELOP/2020/190637

MS TITLE: Regulators of H3K4 methylation mutated in neurodevelopmental disorders control axon guidance in C. elegans.

AUTHORS: Steffen Abay-Noergaard, Benedetta Attianese, Laura Boreggio, and Anna Elisabetta Salcini

© 2020. Published by The Company of Biologists under the terms of the Creative Commons Attribution License (https://creativecommons.org/licenses/by/4.0/).

ARTICLE TYPE: Research Article

I am happy to tell you that your manuscript has been accepted for publication in Development, pending our standard ethics checks.

© 2020. Published by The Company of Biologists under the terms of the Creative Commons Attribution License (https://creativecommons.org/licenses/by/4.0/).
